# Zinc Finger Homeobox Transcription Factors OsMIF1 and OsMIF2 Regulate Grain Size and Panicle Development in Rice

**DOI:** 10.1186/s12284-026-00898-5

**Published:** 2026-03-10

**Authors:** Jinpyo So, Kyoungwon Cho, Jong-Yeol Lee, Don-Kyu Kim, Oksoo Han

**Affiliations:** 1 Department of Molecular Biotechnology and Department of Integrative Food, Bioscience and Biotechnology (BK21 FOUR), Kumho Life Science Laboratory, Chonnam National University, Gwangju, 61186 South Korea; 2 Department of Agricultural Biotechnology, National Institute of Agricultural Science, RDA, Jeonju, 54874 Republic of Korea

**Keywords:** Rice, Zinc finger-homeobox, Mini zinc finger, OsMIF1, OsMIF2, Grain size, Panicle development

## Abstract

Mini zinc finger (MIF) proteins are plant-specific zinc finger-homeodomain (ZF-HD) transcription factors lacking a homeodomain, and its biological functions are critical for normal plant development and the response to environmental stress. Here, CRISPR-Cas9 approach was used to engineer null alleles of rice OsMIF1 and OsMIF2, and the resulting OsMIF1 and OsMIF2 edited lines were used to identify the biological roles of OsMIF1 and OsMIF2. The results suggest that OsMIF proteins regulate grain size by controlling the size of epidermal cells and the length and branching of rice panicles. RNA-seq analysis of OsMIF-knockout cells revealed altered expression of genes involved in development, the response to environmental stress and grain size. In addition, 10 protein-interacting partners of OsMIF1 were identified using a yeast two-hybrid screen: these proteins play roles in diverse developmental, hormonal, stress response, and metabolic processes. The results support the involvement of OsMIF1 and OsMIF2 in the regulation of panicle and grain development and suggest that they may be associated with diverse biological pathways through interactions with multiple regulatory proteins.

**Supplementary Information:**

The online version contains supplementary material available at 10.1186/s12284-026-00898-5.

## Background

Rice is a critically important agricultural crop that is the primary dietary staple and source of calories for nearly half of the human population (Fukagawa & Ziska 2019; Mohidem et al. 2022). Therefore, the quality and quantity of every rice harvest is critical and ways to increase rice yield and quality are a major focus of ongoing agricultural research (Alam et al. 2024; Hori & Sun 2022). Panicle and seed number, architecture/structure, size and composition (Gengmi Li et al. 2021a, b; Gangling Li et al. 2021a, b), which are regulated by a complex network of genetic, hormonal, and environmental factors, are key determinants of rice yield and quality. Moreover, the regulation of these critical rice traits is intricately and coordinately influenced by resource allocation (Gengmi Li et al. 2021a, b; Li et al. 2022; Ren et al. 2023) as well as phytohormones (*i.e.*, abscisic acid (ABA), auxin, cytokinin, and ethylene) and MAPK and G-protein signaling pathways and relevant transcription factors (TFs) (Gengmi Li et al. 2021a, b; Zhao et al. 2022).

TFs play pivotal roles in integrating and coordinating hormonal and other signaling pathways that regulate rice grain and panicle quality and quantity (Gengmi Li et al. 2021a, b; Zhao et al. 2022). For instance, OsSPL family TFs regulate rice panicle architecture and grain traits (Dai et al. 2018; Hu et al. 2021; Lian et al. 2020; Segami et al. 2017; S. Wang et al. 2015a, b), while WRKY TFs orchestrate and integrate both developmental processes and stress response-related pathways and their impact on rice grain and panicle traits (Li et al. 2025; Yang et al. 2025).

The zinc finger-homeodomain (ZF-HD) protein family is a group of plant-specific TFs that regulate plant growth, development, flowering and the response to environmental stressors (Bollier et al. 2022; Jang et al. 2023; Shalmani et al. 2019). ZF-HD proteins harbor both zinc finger (ZF) and homeodomain (HD) motifs, while mini zinc finger (MIF) proteins (a subfamily of ZF-HD proteins) lack the HD motif (Bollier et al. 2022; Islam et al. 2022; Niu et al. 2021). In general, ZF-HD TFs bind to specific cis-acting regulatory motifs in gene promoter regions as monomeric, homo-/hetero- dimeric or higher order protein complexes (Bollier et al. 2022; Shen et al. 2025). These TFs are known to be involved in the regulation of gene expression associated with various biological processes, including organogenesis, hormone signaling, and responses to abiotic and biotic stresses. (Bollier et al. 2022; Shen et al. 2025).

*Arabidopsis* ZF-HD TFs play diverse development-related roles in hormone signaling and light-mediated morphogenesis (Bueso et al. 2014; Kim et al. 2019; Perrella et al. 2018); for example, ZHD5 promotes shoot regeneration and cytokinin-associated phenotypes, ATHB25 (also known as ZFHD2/ZHD1) regulates gibberellin biosynthesis and seed longevity (Bueso et al. 2014; Kim et al. 2019), and ZFHD10 coordinates and integrates light signaling to promote elongation of hypocotyls (Perrella et al. 2018). Rice ZF-HD genes play significant roles in reproductive development including floral initiation and seed formation (Jain et al. 2008; Shalmani et al. 2019; Shen et al. 2025); for example, OsZHD1 and OsZHD2 redundantly regulate grain size, modulate cell proliferation in spikelets and glumes, OsZHD2 also regulates biosynthesis of ethylene which in turn regulates root development (Guo et al. 2025; Yoon et al. 2020), while OsZHD1, OsZHD2, OsZHD4, and OsZHD8 form homo- and heterodimers that directly repress transcription of target genes in plants/cells in the presence of abiotic stressors (Figueiredo et al. 2012).

MIF proteins, which possess only a single zinc finger domain (Hu & Ma 2006; Thiaw & Gantet 2024), play roles in cell division, meristem state transitions, and reproduction-related processes (*i.e.*, development of vegetative and floral organs) (Thiaw & Gantet 2024). In *Arabidopsis*, MIF1 integrates multiple phytohormone signals, and its overexpression results in developmental defects such as dwarfism, loss of apical dominance, and dark-green spoon-shaped cotyledons (Hu & Ma 2006). Furthermore, overexpression of MIF1 or MIF3 induces ectopic shoot meristems along leaf margins, disrupts leaf growth, and modulates auxin and gibberellin-regulated processes (Hu et al. 2011). The tomato MIF homolog SlIMA regulates floral meristem termination, carpel number and fruit development (Bollier et al. 2018), while the *Gerbera hybrida* MIF protein GhMIF directly activates expression of the GEG gene, a member of the GASA family, to suppress ray petal elongation (Han et al. 2017).

A recent study demonstrated that PASPRO1 (OsMIF3) and PASPRO2 (OsMIF4) interact with and inhibit the transcriptional function of rice ZHD TFs, thereby regulating the surface material patterns of the production of anther and pollen. OsMIF3- and OsMIF4 edited lines exhibit abnormal cuticle formation, defective pollen surface structures and reduced fertility (Jang et al. 2023). Furthermore, overexpression of OsMIF1 enhances drought tolerance by regulating developmental processes and interacting with ZHD TFs and OsDIP1, thereby improving resilience of plants exposed to stress (Thiaw 2024). Many studies support a role for MIFs in reproductive development. Despite their potential importance, the biological roles of rice MIFs remain poorly explored.

In this study, CRISPR-Cas9 gene editing was used to generate and then characterize OsMIF1 and OsMIF2 knockout (KO) plants and to elucidate the biological roles of these rice TFs. These studies revealed that OsMIF1 and OsMIF2 KO plants produce enlarged seeds and aberrant panicles. Furthermore, RNA-seq data showed that the phenotypic changes in the engineered KO plants correlate with altered patterns of gene pathway expression. Interestingly, library-scale yeast two-hybrid (Y2H) screening identified 10 candidate protein-interacting partners of OsMIF1; these proteins may provide clues to molecular processes associated with OsMIF1 function.

## Methods

### Plant Materials and Growth Conditions

Seeds of *Oryza sativa* L. ssp. japonica cv. Ilmi were obtained from the Korea Seed & Variety Service. The rice plants were cultivated in experimental paddy fields located in Gwangju, Republic of Korea, during the normal rice-growing season (May–October). Field-grown plants were maintained under conventional agronomic management practices, including basal fertilization prior to transplanting and top dressing according to local recommendations, with continuous flooding irrigation typical for paddy rice cultivation. Standard pest and weed management practices were applied as required.

For propagation and experimental analyses, plants were grown in a controlled plant growth chamber maintained at approximately 28 °C during the day and 22 °C at night, under a 12–14 h light photoperiod, depending on the experimental purpose.

### Sequence Alignment and Prediction of Protein Structure

Fifteen Zinc Finger-Homeodomain (ZF-HD) family proteins were selected as described previously (Hu et al. 2008). The corresponding DNA and protein sequences were obtained from the MSU Rice Genome Annotation Project (MSU RGAP; https://rice.uga.edu/). Protein sequences were aligned using Clustal Omega (https://www.ebi.ac.uk/Tools/msa/clustalo/), and visualized with ESPript (https://espript.ibcp.fr/ESPript/ESPript/). A phylogenetic tree was constructed from the aligned sequences using the Maximum Likelihood method with 1,000 bootstrap replications in MEGA12.

Motif analysis was performed using InterPro (https://www.ebi.ac.uk/interpro/) to predict conserved domains and functional motifs. In addition, the MEME suite (https://meme-suite.org/meme/tools/meme) was used to identify conserved motifs among the ZF-HD proteins. Sequence logos generated by MEME were used to illustrate the positional conservation and amino acid composition of the predicted motifs.

To further investigate structural features, the three-dimensional structures of ZF-HD proteins were predicted using AlphaFold (https://alphafold.ebi.ac.uk/). The predicted protein models were subsequently visualized and analyzed using PyMOL to examine structural features such as conserved domains and secondary structures.

### Subcellular Localization Analysis

For subcellular localization analysis, the full-length coding sequence of *OsMIF1* without the stop codon was amplified using gene-specific primers listed in Additional file 1: Table S1. The PCR product and the p35S-RED-NOS vector were digested with BamHI-HF (Cat. # R3136, New England Biolabs, Ipswich, MA, USA) and KpnI-HF (Cat. # R3142, New England Biolabs, Ipswich, MA, USA), and ligated using the Instant Sticky End Ligase Master Mix (Cat. #M0370, New England Biolabs, Ipswich, MA, USA) according to the manufacturer’s instructions to generate the OsMIF1–RFP fusion construct. The resulting plasmid expressed OsMIF1 in-frame with RFP under the control of the CaMV 35S promoter.

Rice protoplasts were isolated and used for transient expression assays. The OsMIF1–RFP construct was introduced into rice protoplasts by polyethylene glycol (PEG)-mediated transformation. After incubation to allow protein expression, nuclear staining was performed by incubating the protoplasts with Hoechst 33,342 diluted 1:1000 in 1 × PBS for 30 min in the dark. Fluorescence signals were then observed using a confocal laser scanning microscope (FV1000, Olympus, Japan).

### RNA Isolation and Quantitative Real-Time PCR (qRT-PCR)

Total RNA was extracted from each rice tissue using a previously published method (Li & Trick 2005). cDNA was synthesized from 1 µg total RNA using the QuantiTect Reverse Transcription Kit (Cat. #205311, Qiagen, Hilden, Germany), following the manufacturer's instructions. Quantitative real-time PCR (qRT-PCR) was performed using the QuantiTect SYBR Green PCR Kit (Cat. #204343, Qiagen, Hilden, Germany) on a Qiagen Rotor-Gene Q real-time PCR cycler. The thermal cycling conditions were as follows: 95 °C for 15 min; 40 cycles of 94 °C for 15 s, 60 °C for 30 s, and 72 °C for 30 s; followed by a melting curve analysis from 72 to 95 °C. *OsUBI5* was used as an internal control gene to normalize gene expression levels (Jain et al. 2006). Relative expression levels were calculated using the 2^–ΔΔCt^ method (Livak & Schmittgen 2001). All primers used for qRT-PCR are listed in Additional file 1: Table S1.

### GUS Staining Assay

Genomic DNA was extracted from 21-day-old seedlings as described by (Dellaporta et al. 1983). A 1.5 kb promoter fragment corresponding to the genomic sequence upstream of the translational start codon (ATG) of the *OsMIF1* gene was amplified from 5 ng of genomic DNA. The PCR product was purified, digested with EcoRI-HF (Cat. #R3101, New England Biolabs, Ipswich, MA, USA) and NcoI-HF (Cat. #R3193, New England Biolabs, Ipswich, MA, USA), and ligated into the pCAMBIA1201 vector that had been digested with the same enzymes, using Instant Sticky-end Ligase Master Mix (Cat. #M0370, New England Biolabs, Ipswich, MA, USA), according to the manufacturer’s protocol.

The construct was introduced into the *Agrobacterium tumefaciens* strain EHA101 and used to transform embryogenic rice calli derived from mature seeds (Kim et al. 2012). Transcription from the cloned *OsMIF1* promoter was then quantified in various tissues harvested from transgenic lines using a previously described GUS reporter staining technique (Dedow et al. 2022). More specifically, tissue samples were pretreated in ice-cold 90% acetone for 5–15 min, incubated in staining buffer [50 mM sodium phosphate buffer (pH 7.2), 2 mM each of K_4_[Fe(CN)_6_] and K_3_[Fe(CN)_6_], 2% Triton X-100, 2 mM X-Gluc] under vacuum for 5 min, and then incubated overnight at 37 °C in the dark. Samples were rinsed in 70% ethanol before storage at 4 ℃ or immediate quantification of GUS activity.

### Hormone Treatment

To investigate hormone responsiveness, leaf discs were prepared from leaves of 28-day-old rice plants. The leaves were excised and cut into uniform leaf discs, which were then floated on a hormone-containing solution in 6-well plates. The leaf discs were immersed in the treatment solution and incubated at room temperature. Samples were immediately frozen in liquid nitrogen and stored at − 70 °C until further analysis.

### Prediction of Cis-acting Regulatory Elements

The 1.5 kb upstream promoter regions of *OsMIF1* and *OsMIF2* were retrieved from the Rice Genome Annotation Project (http://rice.plantbiology.msu.edu/). Cis-regulatory elements within these promoter sequences were predicted using the PlantCARE database (http://bioinformatics.psb.ugent.be/webtools/plantcare/html/) (Lescot et al. 2002). For visualization, the PlantCARE results were processed and visualized in R (version 4.5.1) using the tidyverse and ggplot2 packages.

### Generation of OsMIF1 and OsMIF2 Knockout Rice

Knockout (KO) lines of OsMIF1 and OsMIF2 were generated in *Oryza sativa* ssp. *japonica* cv. Ilmi through the CRISPR-Cas9 system. A single sgRNA (5′-GCGGCCAAGCCGTACGCGAACGG-3′) was designed using CRISPR-P 2.0 (http://crispr.hzau.edu.cn/CRISPR2/) to target a conserved sequence shared by both *OsMIF1* and *OsMIF2.* The sgRNA was first cloned into the pRGE31 entry vector and subsequently transferred into the pCAMBIA-Cas9 binary vector containing the Cas9 expression cassette and the rice U3 promoter for Agrobacterium-mediated transformation (Chandra et al. 2023; Pham et al. 2024). The pCAMBIA-Cas9-sgRNA construct was introduced into rice via *Agrobacterium tumefaciens* strain EHA101. Agrobacterium-mediated transformation was performed according to a previously described method (Kim et al. 2012).

### Screening and Characterization of KO Mutant Rice Plants

To verify mutations at the target site in T_0_ and T_1_ generations, genomic DNA was extracted from 30-day-old rice plants following a previously described protocol (Dellaporta et al. 1983). The target region was amplified by PCR using specific primers (Additional file 1: Table S1). The resulting amplicons were subjected to deep sequencing using the MiniSeq platform (KAIST, Daejeon, Republic of Korea). The sequencing data were analyzed using RGEN Tools (http://www.rgenome.net). For the T_2_ generation, PCR was performed using specific primers (Additional file 1: Table S1). The amplified products were purified and subjected to Sanger sequencing at Cosmogenetech (Seoul, Republic of Korea) using the BigDye® Terminator v3.1 Cycle Sequencing Kit, and analyzed on an Applied Biosystems 3730xl DNA Analyzer.

### Scanning Electron Microscopy (SEM)

To examine the cell size of mature rice spikelets, scanning electron microscopy (SEM) was performed using a JSM-IT300 (JEOL Ltd., Tokyo, Japan) (Pathan et al. 2008, 2010). Samples were mounted on aluminum stubs, sputter-coated with a thin layer of platinum, and observed under SEM. The length and width of epidermal cells on the outer surface of spikelet hulls were measured using ImageJ software (https://imagej.net/).

### Transcriptome Analysis

Total RNA extracted from immature seeds (14 days after flowering) and developing panicles (10 cm) of non-transgenic (NT) and OsMIF1 and OsMIF2 KO mutant lines was sent to DNALINK Biotechnology Company (Seoul, Republic of Korea) for RNA sequencing. For each genotype and tissue, seeds were collected from three independent plants, and five seeds were harvested from each plant. A total of 15 seeds were pooled and used as one biological sample for RNA extraction and library preparation. This pooled-sample strategy was applied to reduce individual plant variation and to obtain a representative transcriptomic profile for each condition.

Libraries were prepared using the TruSeq Stranded mRNA Sample Preparation Kit (Illumina Inc., San Diego, CA, USA) and sequenced on the Illumina NovaSeq 6000 platform with paired-end reads (2 × 101 bp). Each sample was assigned a unique barcode index, and sequencing was performed according to the manufacturer’s instructions.

Transcript quantification was performed using Kallisto, which performs pseudo-alignment of reads to the reference transcriptome and estimates transcript abundance (Bray et al. 2016). The resulting expression matrix was normalized using TMM (trimmed mean of M-values) normalization. Differentially expressed genes (DEGs) between KO and control samples were identified using both the edgeR and DESeq2 packages in R. Only genes that were consistently identified as DEGs by both methods were considered for downstream analyses. Statistical significance was determined using a threshold of *p* < 0.05 (Love et al. 2014; McCarthy et al. 2012).

Venn diagrams for DEG comparisons among the mutants were constructed using the online tool Venny 2.1.0 (https://bioinfogp.cnb.csic.es/tools/venny/). Gene Ontology (GO) and Kyoto Encyclopedia of Genes and Genomes (KEGG) enrichment analyses were performed using the functional annotation tool DAVID (Database for Annotation, Visualization, and Integrated Discovery, https://davidbioinformatics.nih.gov). GO and KEGG terms with *p*-value ≤ 0.1 were considered significant. For visualization, the data were imported into R (v4.5.1), and bar plots were generated using the ggplot2, dplyr, and forcats packages. Heatmaps for visualization of gene expression patterns were generated using Heatmapper (http://www.heatmapper.ca/expression/), an online tool for expression data clustering and heatmap construction.

Protein–protein interaction networks were predicted using the STRING database (https://string-db.org/). The corresponding protein identifiers were retrieved and queried against the *Oryza sativa* Japonica Group database in STRING. Interactions were filtered using a minimum required interaction score of 0.4, and the resulting network was visualized in Cytoscape for clustering and pathway annotation. KEGG pathway enrichment of the mapped proteins was assessed using STRING’s built-in functional enrichment tool.

### Y2H Library-Scale Screening Assay

Yeast Two-Hybrid screening of OsMIF1 was conducted using the *Saccharomyces cerevisiae* strain AH109, which contains two reporter genes, HIS3 and ADE2, under the control of distinct GAL promoters. The full-length *OsMIF1* was amplified using gene-specific primers containing *Eco*RI and *Bam*HI sites (Additional file 1: Table S1), and cloned into the corresponding sites of the pGBKT7 vector for expression as a myc-tagged GAL4 DNA-binding domain fusion. The construct was confirmed by DNA sequencing and subsequently co-transformed into yeast together with a rice whole-plant cDNA activation domain (AD) library. The cDNA inserts of the library were cloned into the EcoRI and XhoI sites of the pAD-GAL4-2.1 vector. Transformants were selected on synthetic dropout (SD) medium lacking leucine, tryptophan, histidine, and adenine (SD-LWHA), which permits the growth of yeast cells expressing interacting bait and prey proteins.

To confirm protein–protein interactions, DNA fragments encoding the prey proteins from 60 initial candidate clones were isolated via PCR or by transformation into *E. coli*. These prey clones were then reintroduced into yeast AH109 along with either the OsMIF1 bait plasmid or an empty bait vector as a negative control. Interactions were assessed based on yeast growth on SD-LWHA selection medium. Candidates were further validated through DNA sequencing and restriction enzyme digestion.

## Results

### Structural and Evolutionary Features of rice ZF-HD proteins OsMIF1 and OsMIF2

**Fig. 1:**
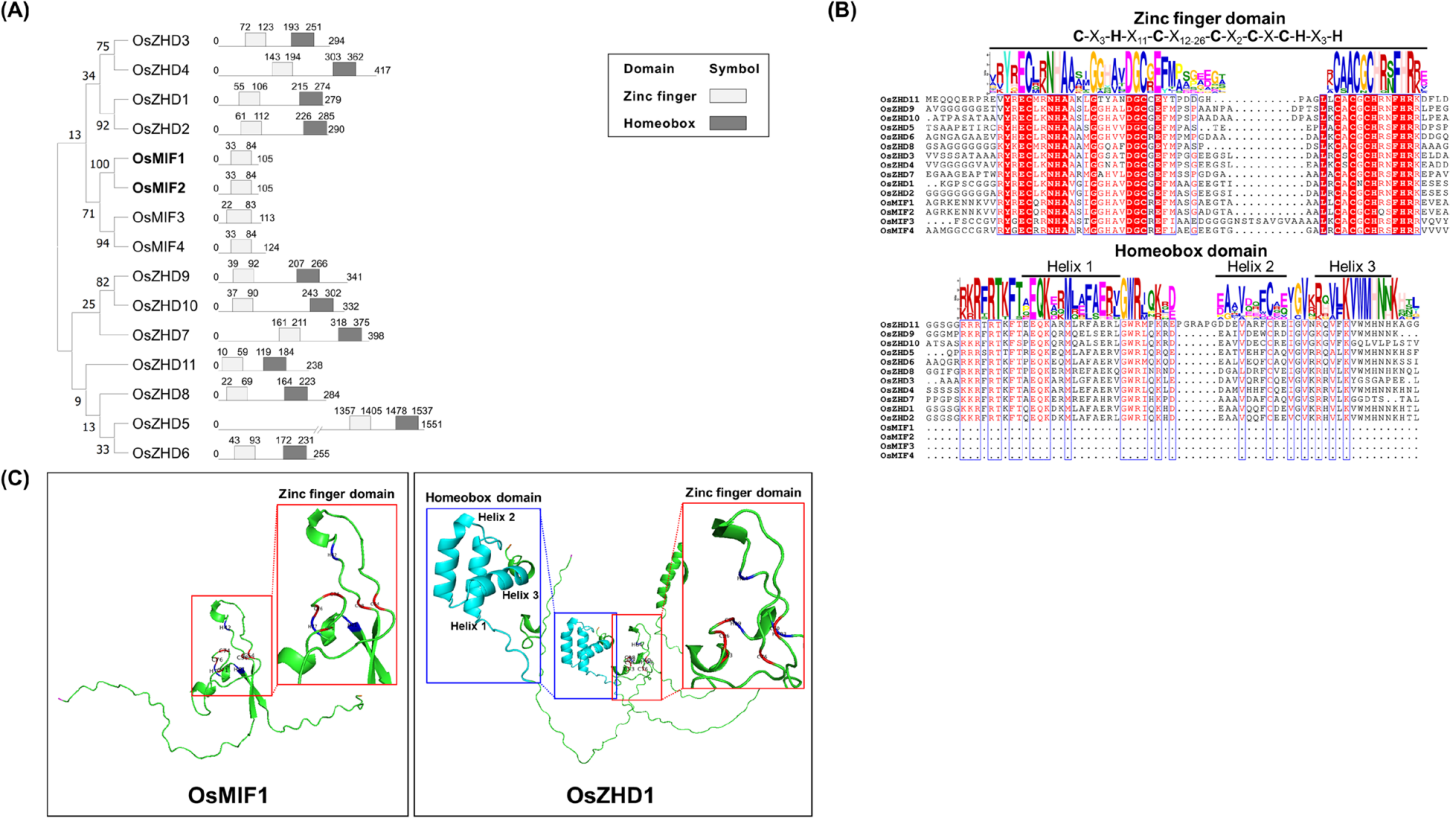
Phylogenetic and Structural Features of Rice ZF-HD Transcription Factors. **A** Phylogenetic tree and domain organization of the ZF-HD family proteins. **B** Multiple sequence alignment showing conserved motifs within the zinc finger and homeobox domains. **C** Predicted 3D structures of OsMIF1 and OsZHD1 proteins

To elucidate the structural characteristics of OsMIF1 and OsMIF2 and determine the percent similarity/divergence among rice ZF-HD proteins, the amino acid sequences of 11 rice ZF-HD and 4 rice MIF proteins were aligned (Additional file 1: Table S2) (Hu et al. 2008) and subjected to phylogenetic tree, domain prediction, and three-dimensional structure prediction analyses (Fig. 1). In the phylogenetic tree for this subset of rice ZF-HDs, OsMIF1 and OsMIF2 clustered together with a bootstrap value of 100%, indicating a highly reliable evolutionary relationship (Fig. 1A). The aligned full-length amino acid sequences of OsMIF1 and OsMIF2 demonstrate 98.1% identity and only two amino acid differences (Fig. 1B). OsMIF3 and OsMIF4 cluster with OsMIF1 and OsMIF2 (bootstrap value 71%) to form a distinct MIF subfamily within the larger rice ZF-HD protein family. OsZHD1–4 are more closely related to OsMIF proteins than to other rice ZF-HD proteins (Fig. 1A). Similar results were obtained when the alignment was limited to the ZF domain in this subset of rice ZF-HD proteins (Additional file 1: Fig. S1).

The domain structure of rice ZF-HDs was predicted using AlphaFold (https://alphafold.ebi.ac.uk/). The results reveal that rice ZF-HD proteins (ZHDs) possess both a zinc finger domain and a homeobox domain, whereas rice MIFs, including OsMIF1 and OsMIF2, possess a zinc finger domain but do not possess a homeobox domain (Fig. 1B, Additional file 1: Table S3). Zinc finger domains contain a conserved C_5_H_3_ motif and homeobox domains are comprised of a characteristic three-helix fold (Fig. 1B). AlphaFold-based 3D structural prediction confirmed that both of these features are present in the rice ZF-HDs (Fig. 1C, Additional file 1: Fig. S2), and that OsMIF1, belonging to the MIF subclass of ZF-HDs, is predicted to lack a homeodomain but to contain a zinc finger domain with the characteristic conserved cysteine (C38, C54, C71, C74, and C76) and histidine (H42, H77 and H81) residues configured for zinc coordination. In contrast, OsZHD1 is predicted to harbor a characteristic zinc finger domain (with conserved cysteines C60, C76, C93, C96, and C98 and histidine residues H64, H99 and H103) and a homeobox with a typical three α-helix domain (Fig. 1C). These results indicate that OsMIF1 and OsMIF2 represent a distinct conserved MIF subgroup in the rice ZF-HD family. In addition, subcellular localization analysis showed that OsMIF1–RFP signals were predominantly detected in the nucleus, indicating that OsMIF1 is a nuclear-localized protein (Additional file 1: Fig. S3).

The bioinformatic tool OrthoDB (https://www.orthodb.org/) identified 65 plant MIF proteins orthologous to OsMIF1 and OsMIF2. Among them, three proteins from wheat (*Triticum aestivum* L.) showed the highest similarity to and formed a distinct subgroup from OsMIF1 and OsMIF2. In addition, eight proteins from maize (*Zea mays* L.) exhibited considerable similarity to OsMIF1 and OsMIF2 (Additional file 1: Fig. S4).

### Tissue- and Stage-specific Expression of OsMIF1 and OsMIF2 During Rice Developmen

**Fig. 2:**
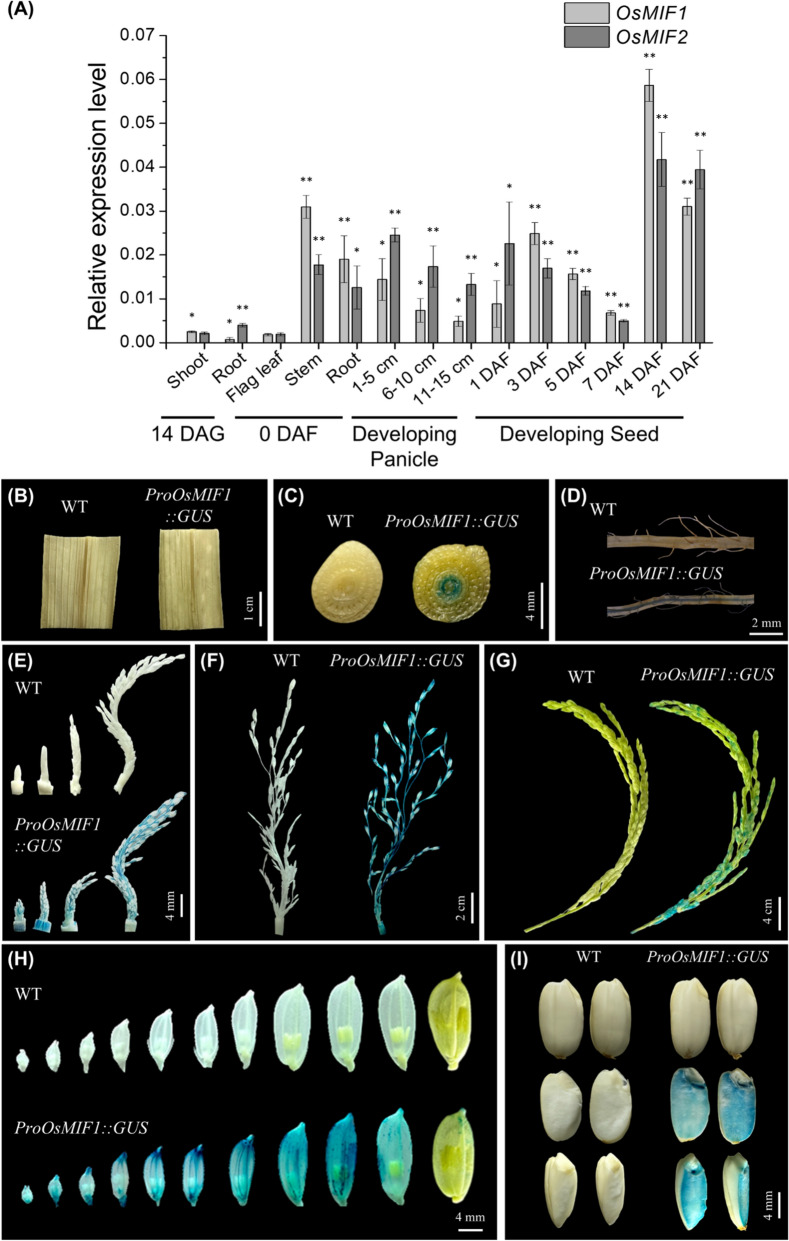
Expression profiling of OsMIF1 and OsMIF2 in various rice tissues. **A** qRT-PCR analysis of *OsMIF1* and *OsMIF2* across different developmental stages and tissues. *OsUBI5* was used as the internal control. Values are mean ± SD (n = 3). *P*-values were calculated using Student’s t-test (*p < 0.1, **p < 0.01). **B–I** Histochemical staining in different tissues of *pOsMIF1::GUS* transgenic rice plant. **B** Flag leaf. **C** Stem. **D** Root. **E–G** Developing panicles. **H** Developing spikelets. **I** Mature seeds

To investigate the tissue- and stage specificity of OsMIF1 and OsMIF2 expression, qRT-PCR and GUS staining assays were performed in diverse tissues of plants at different stages of development (Fig. 2). qRT-PCR data indicate that OsMIF1 and OsMIF2 are expressed at a low level in 14 DAG seedlings but are expressed at a high level in developing panicles before flowering and at an early developmental stage (1–5 cm); their expression then decreased gradually as panicle development progressed. At 0 DAF, their expression was high in stems and roots but low in flag leaves, while *OsMIF1* peaked at 3 DAF and *OsMIF2* peaked at 1 DAF during seed development and both declined from their peak expression level until 7 DAF. Expression of both genes increased from 7 to 14 DAF, and comparable expression levels were observed at 21 DAF (Fig. 2A).

To examine the tissue- and developmental stage-specific expression pattern of OsMIF1, a 1,500 bp fragment of the upstream promoter region of the *OsMIF1* gene was cloned into a β-glucuronidase (GUS) reporter gene vector, and GUS expression was examined in various tissues and at different developmental stages of rice (Fig. 2B–I, Additional file 1: Fig. S5–6). Because the promoter regions of *OsMIF1* and *OsMIF2* show high sequence similarity (94.76%), the *OsMIF1* promoter was used as a representative for GUS staining analysis. The results showed that OsMIF1 expression is not detected in flag leaves at 0 DAF (Fig. 2B), but is expressed in the inner vascular tissues of the stem and root at 0 DAF (Fig. 2C–D). Furthermore, the pattern of GUS reporter gene expression suggests that OsMIF1 is expressed from early developmental stages (0–1 cm) to the intermediate stages (5–10 cm) of panicle development (Fig. 2E–G). The GUS reporter gene expression in spikelets was observed at the distal end from the early to the intermediate developmental stages, became more broadly distributed during the late development stage, and was no longer detected after spikelet growth was completed (Fig. 2H). During seed development, reporter gene expression was initially concentrated at the distal end and later became distributed throughout the entire seed at the middle stages of development (Additional file 1: Fig. S6). In mature seeds, no OsMIF1 expression was detected in the surface cells, whereas OsMIF1 expression was clearly observed in the endosperm cells (Fig. 2I).

Analysis of the 1.5 kb promoters of OsMIF1 and OsMIF2 using PlantCARE revealed the presence of multiple cis-acting regulatory motifs (*i.e.*, hormone-regulating motifs ABA, MeJA and GA; motifs that mobilize responses to stressors including drought, hypoxia, wounding) (Additional file 1: Fig. S7). Transcripts of *OsMIF1* and *OsMIF2* were also quantified in hormone-treated leaf discs using qRT-PCR (Additional file 1: Fig. S8). The results showed that *OsMIF1* is induced by ABA and NAA but suppressed by MeJA and GA_3_, while *OsMIF2* is slightly induced by ABA and NAA and repressed by MeJA and ACC (Additional file 1: Fig. S8).

Overall, these results indicate that OsMIF1 and OsMIF2 are expressed across diverse tissues during reproductive stages, implying their putative roles in panicle development and seed maturation. The fact that transcription of *OsMIF1* and *OsMIF2* is induced/repressed in response to plant hormones suggests that they may be part of hormone-responsive regulatory processes.

### Phenotypic Characterization of OsMIF1, OsMIF2 and OsMIF1/OsMIF2 Mutant Lines

**Fig. 3:**
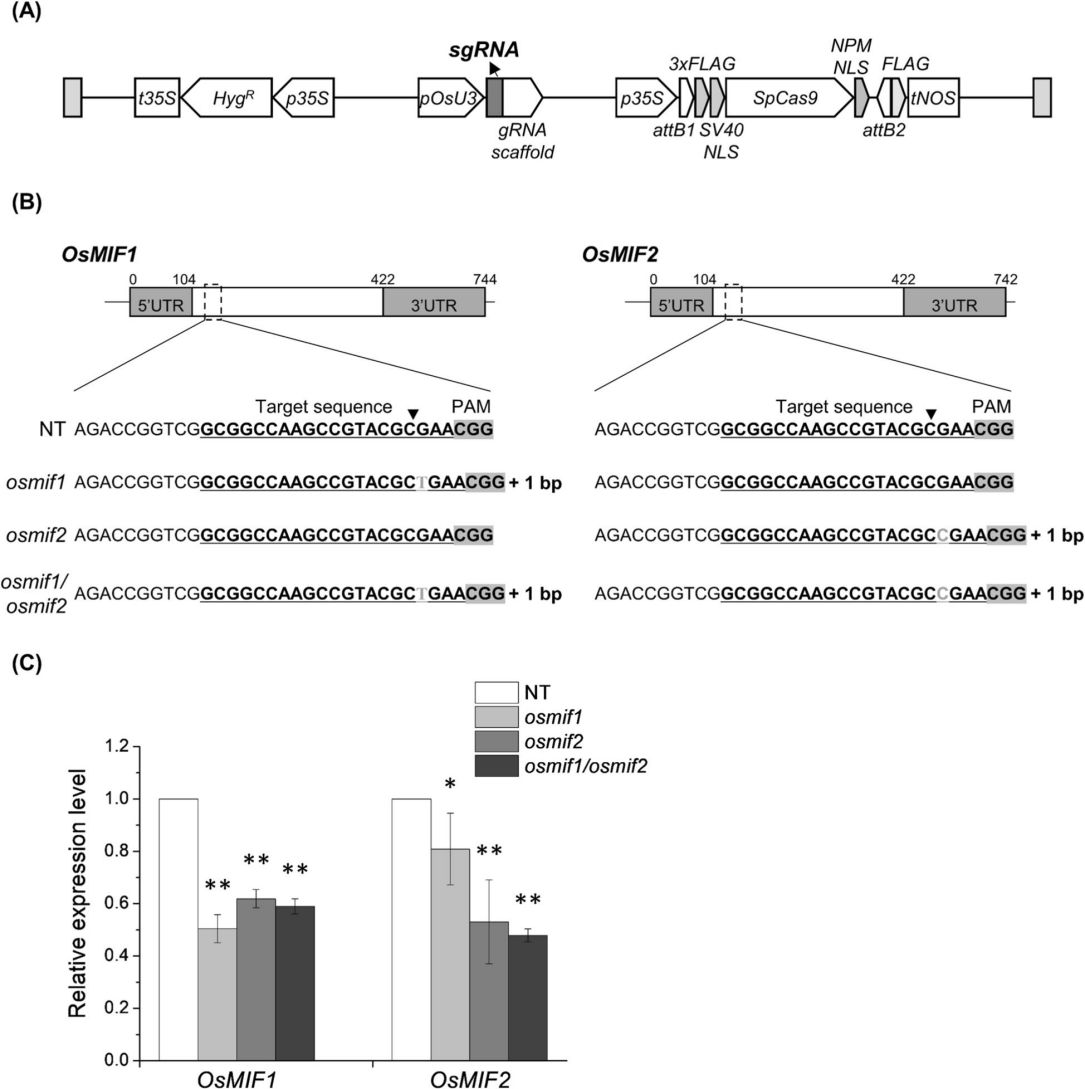
Generation of OsMIF1 and OsMIF2 knockout (KO) lines using CRISPR-Cas9 system. **A** Schematic representation of the CRISPR-Cas9 binary vector used for genome editing. **B** Identification of mutations in T_2_ OsMIF1 and OsMIF2 KO lines. **C** qRT-PCR analysis of *OsMIF1* and *OsMIF2* expression levels in the KO lines compared to the non-transgenic (NT) control. *OsUBI5* was used as the internal control. Error bars represent SD (n = 3). *P*-values were calculated using Student’s t-test (*p < 0.1, **p < 0.01)

CRISPR-Cas9 technology was used to generate OsMIF1 and OsMIF2 knockout (KO) plant lines, which were then used to elucidate the functional roles of these rice MIFs (Fig. 3). Three mutant lines were generated and selected for further study: *osmif1* harbors a 1 bp insertion mutation, *osmif2* also harbors a 1 bp insertion mutation, while the third line, *osmif1/osmif2*, is a double mutant harboring a 1 bp insertion in each *CRISPR-Cas9-*edited MIF gene (Fig. 3B, Additional file 1: Table S4–6). Genomic DNA sequencing confirmed the presence of CRISPR-induced mutations at the target sites in all mutant lines. Consistently, cDNA sequencing revealed that the same mutations were retained at the transcript level. These mutations cause frameshifts that are predicted to alter the amino acid sequence and prevent the formation of the zinc finger domain, which is the major functional domain of OsMIF1 and OsMIF2 (Additional file 1: Fig. S9). Meanwhile, qRT-PCR analysis showed that the transcript levels of OsMIF1 and OsMIF2 were reduced in the mutant lines compared with the wild-type (Fig. 3C).

**Fig. 4:**
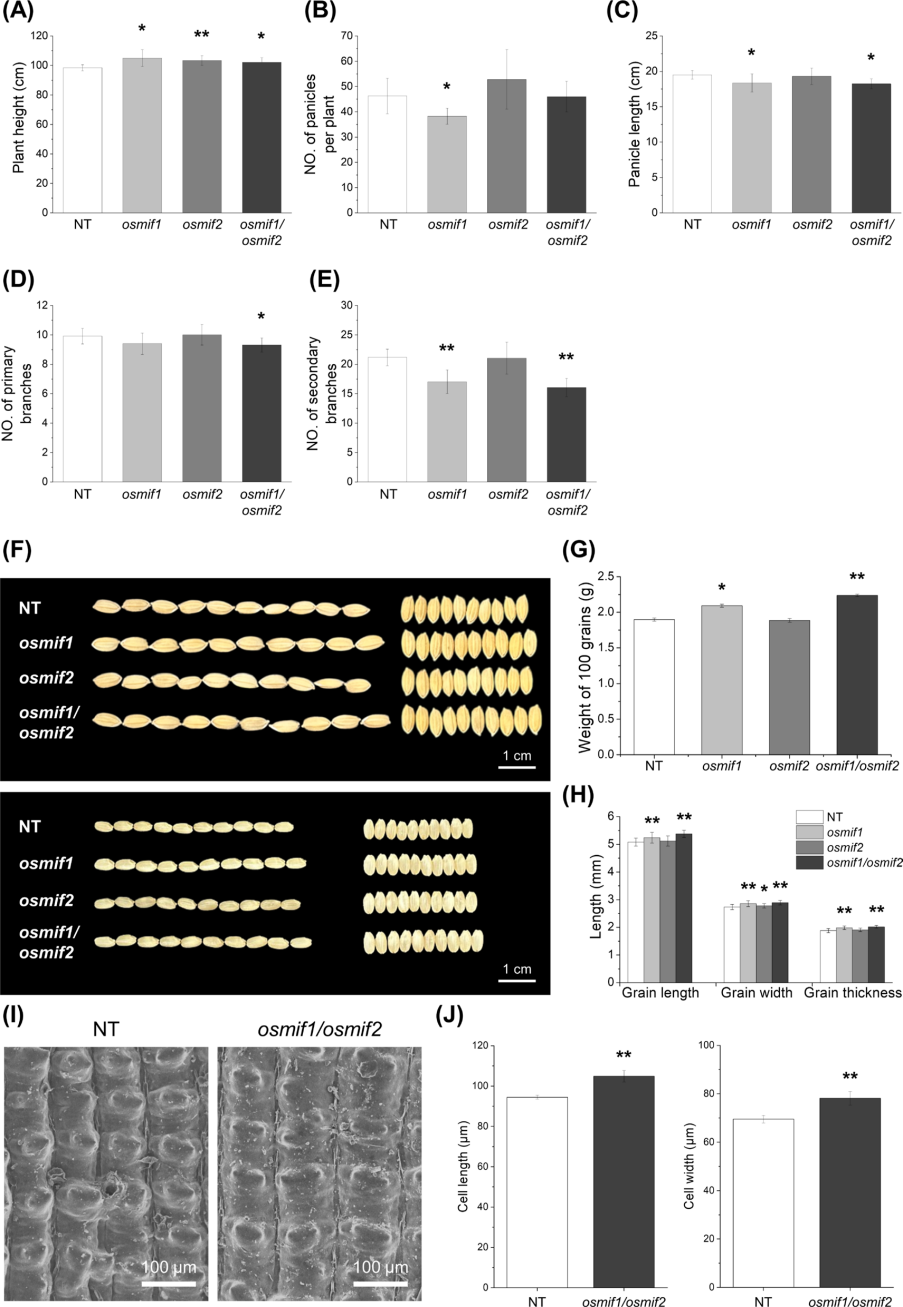
Phenotypic characterization of panicle and grain morphology in *osmif1*, *osmif2*, and *osmif1/osmif2* KO lines. **A–E** Morphological traits of panicles in the NT and KO mutants. **A** Plant height, **B** Number of panicles per plant, **C** Panicle length, **D** Number of primary branches, and **E** Number of secondary branches. Values are mean ± SD (3 ≤ n ≤ 6). **F–H** Seed morphology analysis in NT and KO mutants. **F** Grains with husk (top) and de-husked grains (bottom), **G** weight of 100 grains, and **H** comparison of grain length, width, thickness. Values are mean ± SD (n = 30). **I–J** Scanning electron microscopy (SEM) analysis of the outer surface of NT and *osmif1/osmif2* KO mutant line spikelet hulls. **I** Representative SEM images of the outer surface of spikelet hulls from the NT and the *osmif1/osmif2* double mutant. **J** Average cell length and width of outer epidermal cells in spikelet hulls (n = 10). *P*-values were calculated using Student’s t-test (*p < 0.1, **p < 0.01)

We compared the morphological traits of panicles in *osmif1*, *osmif2*, *osmif1/osmif2*, and wild-type control (NT) plants to test the prediction that the mutant plants would exhibit defects during reproductive stages (Fig. 4A–E, Additional file 1: Fig. S10). Plant height increased in all mutant lines relative to NT, with *osmif1*, *osmif2*, and *osmif1/osmif2* plants being on average 6.7, 5.1, and 3.9% taller than NT, respectively (Fig. 4A). The number of panicles per plant decreased in *osmif1* relative to NT by 17.3%, while the number of panicles per plant was similar in NT, *osmif2* and *osmif1/osmif2* plants (Fig. 4B). Panicles were 5.9% and 6.5% shorter in *osmif1* and *osmif1/osmif2* plants but only slightly shorter in *osmif2* than in NT plants (Fig. 4C). In addition, there were 6.1% fewer primary branches in *osmif1/osmif2* plants than in NT plants (Fig. 4D), and the number of secondary branches was 19.5 and 24.1% lower in *osmif1* and *osmif1/osmif2* plants than in NT plants, respectively. In contrast, primary branching was comparable in *osmif1*, *osmif2* and NT plants, and secondary branching was similar in *osmif2* and NT controls (Fig. 4D and E). These results reveal defects in panicle development, including reduced panicle elongation and branching, in *osmif1* mutant plants.

Our results also provide evidence that OsMIF1/2 influence seed development and morphology (Fig. 4F–J). For example, 100-grain weight of *osmif1* and *osmif1/osmif2* seeds was 10.3% and 17.9% higher than NT seeds, respectively, while 100-grain weight of *osmif2* seeds was 0.6% lower than NT seeds. Consistent with these data, *osmif1* seeds were 3.2% longer, 4.5% wider, and 5.3% thicker than NT seeds, while *osmif2* seeds showed relatively minor (0.8 to 1.6) differences from NT and *osmif1/osmif2* double mutant seeds diverged even more strongly from the control seed, being 5.9% longer, 5.7% wider, and 7.2% thicker than NT seed (Fig. 4F–H). When the outer surface of the spikelets of NT and *osmif1/osmif2* seeds were examined by scanning electron microscopy (SEM), the results show that the epidermal cells of *osmif1/osmif2* double mutant seeds were 12.4% wider and 11.0% longer than NT epidermal cells (Fig. 4I–J). Therefore, *osmif1*, *osmif2* and especially *osmif1/osmif2* plant lines exhibit increased seed size and weight, which is consistent with the observed increased size of plant epidermal cells.

The phenotypic analyses indicate that OsMIF1 plays a predominant role in reproductive development, as knockout of OsMIF1 alone resulted in clear and statistically significant phenotypic changes, whereas knockout of OsMIF2 alone caused limited or no significant effects. Moreover, the *osmif1/osmif2* double knockout plants exhibited more pronounced phenotypes than the *osmif1* single mutant. Together, these results suggest that OsMIF1 functions as a primary regulator, while OsMIF2 acts as a secondary and supportive factor. Overall, our findings suggest that OsMIF1 and OsMIF2 cooperatively contribute to reproductive development by influencing panicle architecture and seed morphology.

### Global Gene Expression Profiling of *osmif1 *and *osmif2* Panicles and Immature Seeds

**Fig. 5:**
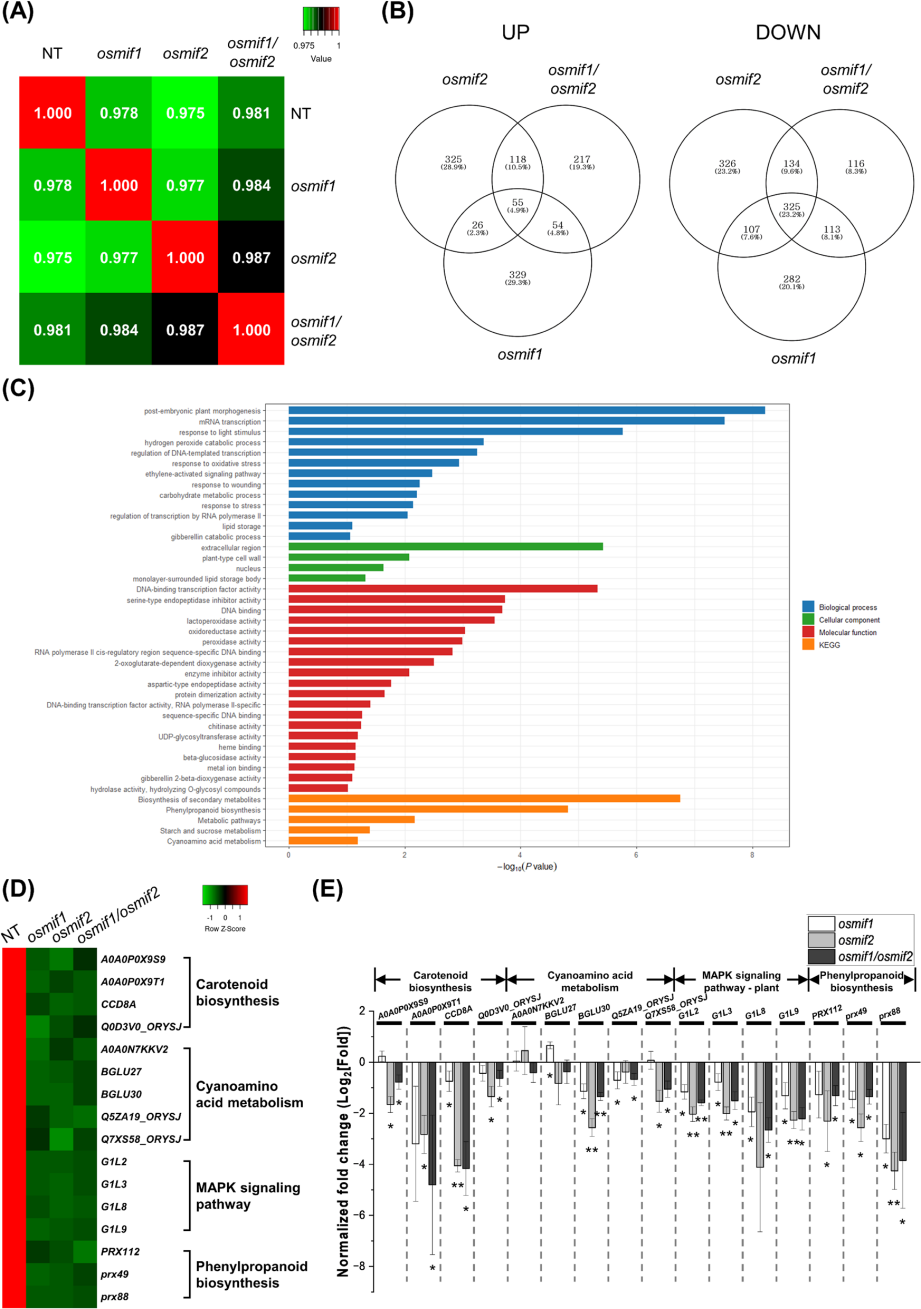
RNA-seq-based transcriptome analysis of 10-cm developing panicles from *osmif1*, *osmif2*, and *osmif1/osmif2* KO plants. **A** Pearson correlation matrix of transcriptomic profiles of NT, *osmif1*, *osmif2*, and *osmif1/osmif2* lines. **B** Venn diagrams of differentially expressed genes (DEGs) that are up-regulated (left) or down-regulated (right) in each KO line (the non-transgenic (NT) line was used as the control/reference line). **C** Gene ontology (GO) classification of down-regulated DEGs using the DAVID tool. **D–E** Expression profiling of DEGs associated with KEGG pathways significantly enriched according to STRING database. **D** Heatmap based on RNA-seq data. Relative expression is represented using normalized Z-score values. **E** qRT-PCR analysis of selected DEGs. Expression of target genes was normalized using the 2^−ΔΔCT^ method; relative expression in each *osmif1* and *osmif2* KO lines using NT as control is shown as log_2_ of the average fold-change using *OsUBI5* as an internal control. Error bars represent SD (n = 3). *P*-values were calculated using Student’s t-test (*p < 0.1, **p < 0.01)

Global transcriptomic analyses were performed to correlate OsMIF1/OsMIF2 genotype with phenotype and associated changes in expression of relevant biological pathways. To this end, RNA-seq data were collected using samples from 10 cm developing panicles and 14 DAF immature seeds from NT, *osmif1*, *osmif2*, and *osmif1/osmif2* plant lines (Fig. 5, Additional file 1: Fig. S11). In 10 cm developing panicles, RNA-seq yielded 39–51 million reads per sample with Q30 values above 91%. In 14 DAF seeds, 25–34 million reads per sample were obtained, and all libraries showed Q30 values above 87% (Additional file 1: Table S7). Correlation coefficients varied from 0.975 to 0.988, indicating that the RNA-seq data were derived from the same developmental stage and tissue (Fig. 5A, Additional file 1: Fig. S11A).

The RNA-seq data were subjected to differential expression analysis using edgeR and DESeq2 software packages (significance threshold was *p* < 0.05). In samples from 10 cm developing panicles, 1,124 genes were upregulated, including 55 genes expressed in all mutant lines, while 1,403 genes were downregulated, of which 325 transcripts were consistently detected independent of the genotype of the sample (Fig. 5B). GO analysis was performed using the DAVID tool (https://davidbioinformatics.nih.gov), which links each up- or down-regulated gene with annotations that assign an established or putative biological function, *e.g.* Biological Process (BP), Cellular Component (CC), Molecular Function (MF), KEGG pathway (Fig. 5C).

GO enrichment analysis of the 325 commonly downregulated genes revealed significant enrichment in a subset of BP terms including post-embryonic plant morphogenesis, mRNA transcription, response to light stimulus, hydrogen peroxide catabolic process, and response to oxidative stress. Enriched CC terms included nucleus, extracellular region, plant-type cell wall, and lipid storage body, while enriched MF terms included DNA-binding transcription factor activity, oxidoreductase activity, and peroxidase activity; enzyme inhibitors were also significantly overrepresented. KEGG analysis revealed enrichment of pathways involved in biosynthesis of secondary metabolites, phenylpropanoid biosynthesis, (general) metabolism, starch and sucrose metabolism, and cyanoamino acid metabolism (Fig. 5C). To validate the RNA-seq data, qRT-PCR was performed for a subset of downregulated DEGs, including those associated with carotenoid biosynthesis, cyanoamino acid metabolism, MAPK signaling, and phenylpropanoid biosynthesis (Fig. 5D–E, Additional file 1: Fig. S12-13, Additional file 1: Table S8–9). Expression values for DEGs were normalized to an appropriate control using the 2^−ΔΔCT^ method and results are presented as log₂ values. The qRT-PCR results validated the downregulation patterns of these representative genes identified by RNA-seq (Fig. 5E). The results indicate that knockout mutations in OsMIF1 and OsMIF2 are associated with differential expression of genes involved in morphogenesis, stress responses, transcriptional and enzymatic activities, and hormone and metabolic pathways, suggesting that OsMIF genes play important regulatory roles in rice development and potentially in stress-related responses.

GO enrichment analysis was also performed on RNA-seq data from immature rice seeds (14 DAF). These data revealed that downregulated DEGs are enriched in the following GO terms: photosynthesis, carbon fixation, cell wall modification, photosystem components, thylakoid membrane proteins, and chloroplast-associated factors (Additional file 1: Fig. S11C). qRT-PCR analysis of photosynthesis-related genes confirmed the prediction that they would be down-regulated in *osmif1* (KO) rice relative to NT controls, thus validating the RNA-seq findings described above (Additional file 1: Fig. S11D–E, Additional file 1: Table S10).

### Functional Roles of OsMIF1 and OsMIF2 during Seed Development

**Fig. 6:**
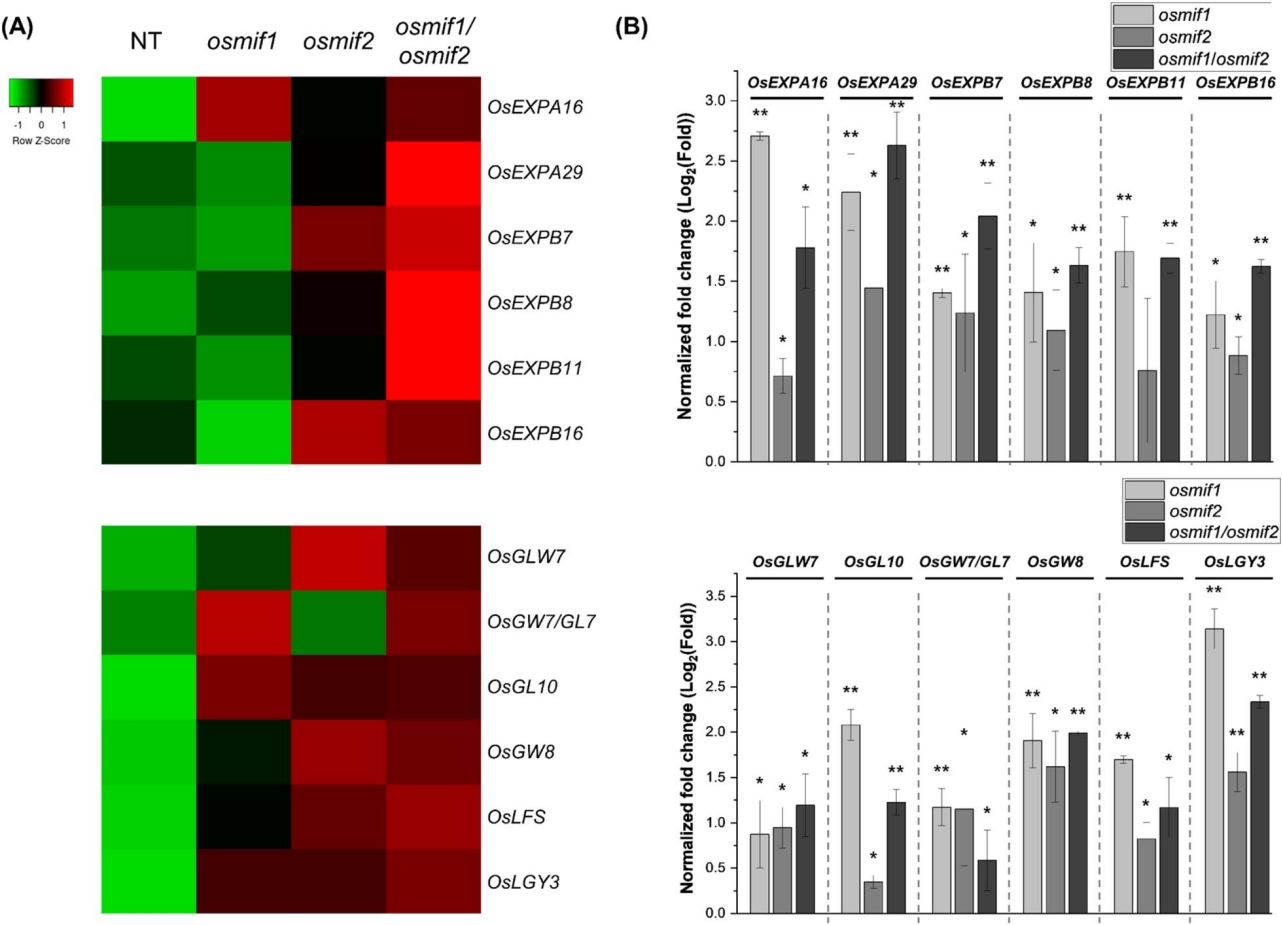
Expression profiling of genes that regulate grain size in 10-cm developing panicles. **A** Heatmap based on RNA-seq data. Expression levels were calculated using TMM-normalized TPM values and are shown using normalized Z-scores. **B** qRT-PCR of genes that regulate cell and grain size. Expression of target genes was normalized using the 2^−ΔΔCT^ method; normalized expression values are represented as log_2_ of the average fold-change using *OsUBI5* as the internal control. Error bars represent SD (n = 3). *P*-values were calculated using Student’s t-test (*p < 0.1, **p < 0.01)

As described above, *osmif1* and *osmif2* KO mutants exhibited increased seed size and enlarged spikelet epidermal cells compared with NT plants (Fig. 4F–J). To gain insight into the molecular basis underlying these phenotypic changes, we examined the expression of selected genes associated with seed size determination and cell expansion (Additional file 1: Table S11) (Lee & Kende 2002; Li et al. 2020; Shim et al. 2022; Shin et al. 2005; Si et al. 2016; S. Wang et al. 2015a, b; Yu et al. 2018; Zhan et al. 2022; Zhou et al. 2015; Zuo et al. 2021). Our RNA-seq data had revealed higher expression of the genes related with seed size determination and cell expansion in the KO mutants than in NT control plants (Fig. 6A); furthermore, qRT-PCR data revealed that all of the corresponding genes are upregulated in the KO mutant lines (Fig. 6B). These results suggest that the increased seed size and enlarged spikelet epidermal cells observed in the *osmif1* and *osmif2* mutants are associated with the upregulation of genes involved in seed size determination and cell expansion.

Previous studies reported that OsMIF1 and OsMIF2 are co-expressed with major seed storage proteins (SSPs) (So et al. 2025). Furthermore, data presented above (Fig. 2) demonstrate that both genes are highly expressed in immature seeds with peak expression at 14 DAF. To further explore potential crosstalk between SSPs (and/or co-expression of SSPs) and OsMIF1/OsMIF2, expression of these genes was quantified and compared in MIF1/2 KO and NT seeds (Additional file 1: Fig. S14). For this analysis, SSP genes were grouped according to their previously reported classification (Additional file 1: Table S12) (Chandra et al. 2023; Pham et al. 2024). First, expression of SSPs was quantified using RNA-seq data from 14-DAF immature seeds. Although no consistent trend was observed in these data, transcripts from the Pro13b-II subgroup of prolamin genes were generally downregulated (Additional file 1: Fig. S14A). This result was confirmed by performing qRT-PCR on Pro13b-II genes using cDNA and primers reported in previous studies (Additional file 1: Fig. S14B) (Pham et al. 2024).

### Identification of Putative OsMIF1 Protein-Interacting Partners by Yeast Two-Hybrid Screening

In the initial discovery of the ZF-HD protein family, Y2H assays revealed that ZF-HD proteins form both homo- and heterodimers (Windhövel et al. 2001). Based on these findings, we conducted a Y2H screen for protein-interacting partners of OsMIF1, in which OsMIF1 was the “bait” and the “prey” were protein products expressed from a whole rice genome cDNA library (Additional file 1: Fig. S15).

The screen identified 18 candidate protein-interacting partners of OsMIF1, of which 10 were in-frame (Additional file 1: Table S13–14). The 10 in-frame proteins included OsSIZ1 (NM_001420071), OsCIPK14 (NM_001404631), akin-beta (XM_015783664), OsMIF1 (NM_001423141), OsBIG (NM_001422705), Snf7 family protein (XM_015782163), OsMORF8b (XM_015756999), OsDjA6 (NM_001402407), OsNBR1 (NM_001416698), and OsELF3.2 (XM_015795402). These putative OsMIF1-interacting proteins are involved in diverse processes, including growth and development, hormone signaling, stress responses, and RNA metabolism (Table 1, Additional file 1: Table S15). One of the interacting partner proteins was OsMIF1 itself, suggesting a possible self-interaction of OsMIF1 (Table 1). This result also predicts the potential for interactions between OsMIF1 and other ZF-HD proteins. GO enrichment analysis revealed that three candidate protein-interacting partners of OsMIF1, *i.e.*, OsSIZ1, OsBIG, and OsDjA6, are predicted to contain a zinc finger motif. Thus, OsMIF1 may interact not only with ZF-HD proteins but also with other ZF proteins (Additional file 1: Fig. S16).

**Table 1:** Candidate OsMIF1-interacting proteins identified by Y2H library screening

No	Gene symbol	NCBI accession number	Function	Reference
1	OsSIZ1	NM_001420071	Regulates growth and development, and mediates responses to phosphate/nitrogen status and environmental stresses	(Mishra et al. 2018, 2017; Park et al. 2010; Thangasamy et al. 2011; Wang et al. 2011a, b, 2015a)
2	OsCIPK14	NM_001404631	Mediates Ca^2^⁺-dependent MAMP-induced defense signaling	(Kurusu et al. 2010)
3	akin-beta	XM_015783664	Is suppressed by OsTZF1 and is induced under cold stress in tolerant rice	(Ding et al. 2025; Jan et al. 2013)
4	OsMIF1	NM_001423141	Enhances drought tolerance by regulating rice growth and development	(Thiaw 2024)
5	OsBIG	NM_001422705	Is essential for rice growth and development; loss of function causes seedling lethality	(Cheng et al. 2019)
6	Snf7 family protein	XM_015782163	Is upregulated in leaf and root under direct-sown drought stress	(Kumar et al. 2022)
7	OsMORF8b	XM_015756999	Acts as a multiple organellar RNA-editing factor interacting with other OsMORFs, and is downregulated by cold and salt stress	(Zhang et al. 2019)
8	OsDjA6	NM_001402407	Negatively regulates rice innate immunity, likely via the ubiquitin–proteasome degradation pathway	(Sarkar et al. 2013; Zhong et al. 2018)
9	OsNBR1	NM_001416698	Mediates pest resistance and enhances cold tolerance via autophagy and reduced ubiquitination	(Guo et al. 2023; Zhang & Chen 2020)
10	OsELF3.2	XM_015795402	Controls heading date, circadian rhythm, and stress tolerance in rice	(Fu et al. 2009; Wang et al. 2021; Zhao et al. 2012)

### Defects in Root Development and the Response to Salt Stress in OsMIF1- and OsMIF2-KO Mutants

**Fig. 7:**
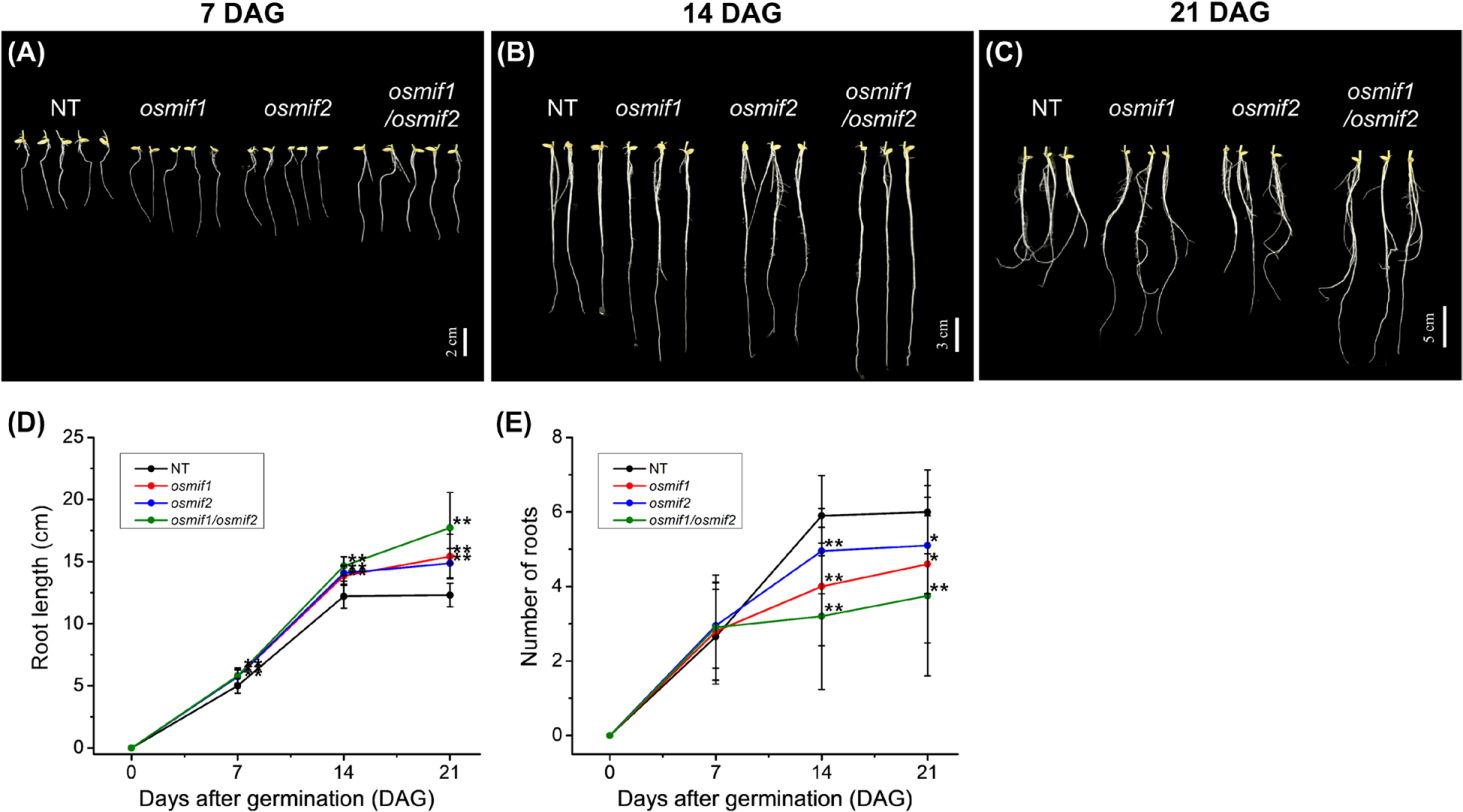
Defects in root morphology and development in young *osmif1* and *osmif2* KO seedlings. **A–C** Representative roots in NT, *osmif1*, *osmif2*, and *osmif1/osmif2* seedlings at 7 days after germination (DAG) (**A**), 14 DAG (**B**), and 21 DAG (**C**). **D** Root length and (**E**) root number in NT and mutant seedlings at 7, 14, and 21 DAG. Error bars represent SD (n = 20). *P*-values were calculated using Student’s t-test (*p < 0.1, **p < 0.01)

The ten-candidate protein-interacting partners of OsMIF1 include proteins associated with the response to drought, salinity and other environmental/water stressors (Table 1). In addition, a previous study reported that overexpression of OsMIF1 affected root growth (Thiaw 2024). These observations suggest that OsMIF1 could influence plant stage-specific changes in root morphology after germination (Fig. 7). Consistent with this hypothesis, phenotypic analysis revealed enhanced root elongation during the early vegetative stage in *osmif1* (KO) plants. Relative to NT controls, *osmif1* plant roots were 14–16% longer at 7 DAG, 14–20% longer at 14 DAG, and 21–44% longer at 21 DAG. Furthermore, this phenotypic trait was exacerbated in roots of *osmif1/osmif2* plants (Fig. 7D). In contrast, root number was slightly (6–11%) higher at 7 DAG, but 16–46% and 18–38% lower than NT controls in 14 and 21 DAG plants. This phenotypic trait was also exacerbated in *osmif1/osmif2* double mutant plants (Fig. 7E).

The effects of salt stress on germination rate were also compared in *osmif1*, *osmif2*, *osmif1/osmif2* and NT control plants. The results showed that NT seeds consistently germinate more rapidly than OsMIF mutant seeds. In the absence of salt stress, NT seeds reached 93.3% germination by 96 h, whereas *osmif1*, *osmif2*, and the *osmif1/osmif2* double mutants achieved 70.0%, 55.2%, and 82.1% germination, respectively. In the presence of 75 mM NaCl, (*e.g.*, conditions of salt stress), germination was 55.2% in NT at 96 h, but only 22.2–26.1% for *osmif1* mutant seeds. Higher salt stress (*i.e.*, 150 mM NaCl) or the *osmif1/osmif2* double mutant genotype exacerbated this phenotype. thus, germination rate was 25.0% for NT seeds at 96 h but 0–5.6% in *osmif1/2* mutants (Additional file 1: Fig. S17), with the lowest germination rate in *osmif1/osmif2* double mutant seeds under high salt stress.

In summary, these results suggest a potential role of OsMIF1 and OsMIF2 in coordinating early root development and in contributing to stress-sensitive germination responses.

## Discussion

### *osmif1/2* KO Genotype Associated with Defects in Reproductive Development

This study exploits transcription profiling and CRISPR-Cas9 gene editing technology to elucidate the biological roles of mini zinc finger TFs OsMIF1 and OsMIF2. Previous studies have shown that plant-specific MIFs play critical roles regulating plant growth and development, hormone signaling, and stress response pathways (Hu et al. 2008; Shen et al. 2025; Tran et al. 2007). More specifically, MIF proteins are critical during reproductive development, regulating floral meristem termination, carpel number, petal elongation, and anther and pollen development (Bollier et al. 2018; Han et al. 2017; Thiaw & Gantet 2024); the mechanisms by which they regulate these processes were not clear.

In rice, there are four MIFs such as OsMIF1 ~ 4 (Hu et al. 2008). A phylogenic tree based on similarity of amino acid sequence showed that OsMIFs are divided into two groups such as OsMIF1/2 and OsMIF3/4 (Fig. 1A). OsMIF3 (PASPRO1) and OsMIF4 (PASPRO2) are specifically expressed in pollen and the anther wall, where they play important roles during the reproductive stage by regulating the surface morphology of anthers and pollen to ensure normal reproductive development (Jang et al. 2023). OsMIF3 and OsMIF4 are not expressed in panicles and seeds, whereas OsMIF1 and OsMIF2 are expressed in panicles, seeds, stems and roots (Fig. 2) (Jang et al. 2023). The amino acid sequences and gene expression patterns suggest that OsMIFs between the two groups play distinct functional roles, but OsMIFs within each group have functional redundancy. In this study, the editing of the OsMIF1 and OsMIF2 genes using CRISPR-Cas9 technology resulted in mutations in the reading frame of each gene, ultimately causing the loss of the zinc finger domains of each protein (Additional file 1: Fig. S9). The mutants showed reduced panicle branching and increased grain size due to increased cell size, and a higher abundance of SSPs reflecting increased transcription of SSP genes (Fig. 3–4, Additional file 1: Fig. S14).

### Prediction of Functional Connections Between MIF and Zinc Finger Proteins

The zinc finger domain analysis of OsMIF and OsZHD proteins (Fig. 1B) showed that they have the same ZF dimerization domain sequences (CX_3_HX_11_CX_12-26_CX_2_CXCHX_3_H), which are involved in protein–protein interaction (Jang et al. 2023; Thiaw & Gantet 2024). Jang et al. (2023) showed that OsMIF3 and OsMIF4 interact with OsZHD1 and OsZHD2, which have similar expression patterns, using yeast two-hybrid assay and bimolecular fluorescence complementation assay, and suggested that the OsMIFs interact with the zinc finger domain in OsZHD transcription factors and regulate their transcription activities.

Rice ZHD proteins OsZHD1 and OsZHD2, which belong to another branch of the ZF-HD family, also share roles in root development and seed size (Guo et al. 2025; Yoon et al. 2020). In contrast to the phenotype of the *osmif1/2* KO mutant including larger seed size (Fig. 4) and longer root length (Fig. 7), the *oszhd1* and *oszhd2* KO mutants were characterized by smaller seed size and shorter root length (Guo et al. 2025; Yoon et al. 2020). ZF-HD proteins tend to homodimerize or form heterodimers (Windhövel et al. 2001). Consistent with this, rice ZF-HD TFs such as OsZHD1, OsZHD2, OsZHD4, and OsZHD8 form homo- or heterodimers (Figueiredo et al. 2012; Guo et al. 2025). RNA-Seq analysis coupled with qRT-PCR showed that the function-loss of OsZHD1 and OsZHD2 alters the expression of cell cycle/expansion and grain-size-related genes (Guo et al. 2025). Additionally, a potential interaction between OsMIF1 and OsZHD proteins was previously reported using a Y2H study (Thiaw 2024). *Arabidopsis* MIFs interact with ZHDs (e.g., ZHD5), inhibiting their nuclear localization and DNA-binding activity (Hong et al. 2011). AtMIF2 and tomato SlIMA directly interact with the C2H2 zinc-finger protein KNU to form a transcriptional repressor complex with corepressors TOPLESS and HDA19. These protein–protein interactions repress expression of WUS/SlWUS, thereby regulating floral meristem termination and carpel number (Bollier et al. 2018). Together the results, the profiling of zinc-finger domain-containing proteins as candidates interacting with OsMIF1 using Y2H library screening (Table 1) and the down-regulation of cell expansion and grain size-related genes in OsMIF1 and OsMIF2 mutants (Fig. 6) indicate that OsMIF1/2 are involved in various biological processes including seed development, and suggest directions for research into the mechanisms and interactions between MIF genes and ZF/ZHD proteins to discover novel protein–protein interactions, elucidate their downstream effects, and gain insights into the structure and activity of plant regulatory networks.

### Potential Roles of OsMIF1 and ZHD Proteins in Root Development

Recent studies have shown that overexpression of OsMIF1 results in impaired root development (Thiaw et al. 2025). Consistently, in our study, mutations in OsMIF1 exhibited a tendency toward increased root length (Fig. 7). In addition, treatment with NAA induced the expression of *OsMIF1* (Additional file 1: Fig. S8), whereas under 2,4-D treatment, the OsMIF1 overexpression lines showed a reduced induction of the auxin-responsive gene *OsIAA9* (Thiaw et al. 2025). These observations imply that OsMIF1 is responsive to auxin signaling while at the same time modulating the transcriptional output of auxin-responsive pathways. Further studies examining the transcriptional regulatory activity and target genes of OsMIF1 in the context of auxin signaling and root development will contribute to a deeper molecular understanding of how OsMIF1 integrates hormonal signals with root growth.

Meanwhile, OsZHD2 has been reported to be associated with root development. OsZHD2 activates ACS genes, thereby increasing ethylene biosynthesis, which in turn promotes auxin accumulation at the root tip and enhances both cell division and cell elongation in the root meristem (Yoon et al. 2020). In *Arabidopsis*, AtZHD5 (HB33), one of the ZF-HD TFs, has been identified as a regulator of primary root growth by mediating the crosstalk between ABA and auxin signaling pathways (Kim et al. 2019; L. Wang et al. 2011a, b). Importantly, the activity of AtZHD5 is negatively regulated by the mini zinc finger protein AtMIF1. AtMIF1 interacts with ZHD5 and forms nonfunctional heterodimers, thereby inhibiting the DNA-binding activity of ZHD5 and attenuating ZHD5-mediated transcriptional regulation (Hong et al. 2011). Consistent with this regulatory mechanism, overexpression of AtMIF1 results in reduced root growth and abnormal root hair development (Hu & Ma 2006). In rice, OsMIF3 and OsMIF4 have been shown to interact with OsZHD1 (Jang et al. 2023), and it has also been suggested that OsMIF1 may interact with OsZHD1 (Thiaw et al. 2025). In addition, our study revealed that the zinc finger domains of OsZHD1 and OsZHD2 exhibit a high degree of sequence similarity.

Taken together, these findings suggest that a conserved MIF–ZHD regulatory module may operate in rice, similar to that described in *Arabidopsis*. This raises the possibility that OsMIF1 and OsMIF2 interact with OsZHD2 to modulate hormone-dependent root development. Furthermore, these observations highlight the need for future studies to investigate the potential interactions among OsMIF1, OsMIF2, and OsZHD2 and to elucidate the molecular mechanisms by which these proteins coordinate ethylene–auxin signaling and root developmental processes.

## Conclusion

This study demonstrates, through CRISPR-Cas9–based genome editing combined with transcriptomic and phenotypic analyses, that OsMIF1 and OsMIF2 are important regulatory factors controlling panicle branching, grain size, and cell expansion during reproductive development in rice. Our results further indicate that they fine-tune transcriptional regulation via protein–protein interaction networks involving ZF/ZHD transcription factors and are functionally linked to plant hormone signaling pathways such as auxin and ethylene, thereby suggesting MIF proteins as potential components of a transcriptional regulatory module coordinating rice growth and development.

## Supplementary Information

Supplementary Material 1.

## Data Availability

No datasets were generated or analysed during the current study.

## Abbreviations

ABA: Abscisic acid
ACC: 1-Aminocyclopropane-1-carboxylic acid
CRISPR-Cas9: Clustered regularly interspaced short palindromic repeats–CRISPR-associated protein 9
DAF: Days after flowering
DAG: Days after germination
DEGs: Differentially expressed genes
GA: Gibberellic acid
GO: Gene ontology
GUS: β-Glucuronidase
HD: Homeodomain
KEGG: Kyoto encyclopedia of genes and genomes
KO: Knockout
MAPK: Mitogen-activated protein kinase
MeJA: Methyl jasmonate
MIF: Mini zinc finger
NAA: 1-Naphthaleneacetic acid
NT: Non-transgenic
SEM: Scanning electron microscopy
SSP: Seed storage protein
TF: Transcription factor
Y2H: Yeast two-hybrid
ZF: Zinc finger
ZF-HD: Zinc finger–homeodomain

## References

[CR1] Alam M, Lou G, Abbas W, Osti R, Ahmad A, Bista S, Ahiakpa JK, He Y (2024) Improving rice grain quality through ecotype breeding for enhancing food and nutritional security in Asia–Pacific Region. Rice 17(1):47. 10.1186/s12284-024-00725-9 39102064 10.1186/s12284-024-00725-9 PMC11300782

[CR2] Bollier N, Sicard A, Leblond J, Latrasse D, Gonzalez N, Gévaudant F, Benhamed M, Raynaud C, Lenhard M, Chevalier C, Hernould M, Delmas F (2018) At-MINI ZINC FINGER2 and Sl-INHIBITOR OF MERISTEM ACTIVITY, a conserved missing link in the regulation of floral meristem termination in *Arabidopsis* and tomato. Plant Cell 30(1):83–100. 10.1105/tpc.17.00653 29298836 10.1105/tpc.17.00653 PMC5810569

[CR3] Bollier N, Gonzalez N, Chevalier C, Hernould M (2022) Zinc finger-homeodomain and mini zinc finger proteins are key players in plant growth and responses to environmental stresses. J Exp Bot 73(14):4662–4673. 10.1093/jxb/erac194 35536651 10.1093/jxb/erac194

[CR4] Bray NL, Pimentel H, Melsted P, Pachter L (2016) Near-optimal probabilistic RNA-seq quantification. Nat Biotechnol 34(5):525–527. 10.1038/nbt.3519 27043002 10.1038/nbt.3519

[CR5] Bueso E, Muñoz-Bertomeu J, Campos F, Brunaud V, Martínez L, Sayas E, Ballester P, Yenush L, Serrano R (2014) *Arabidopsis thaliana* HOMEOBOX25 uncovers a role for gibberellins in seed longevity. Plant Physiol 164(2):999–1010. 10.1104/pp.113.232223 24335333 10.1104/pp.113.232223 PMC3912122

[CR6] Chandra D, Cho K, Pham HA, Lee JY, Han O (2023) Down-regulation of rice glutelin by CRISPR-Cas9 gene editing decreases carbohydrate content and grain weight and modulates synthesis of seed storage proteins during seed maturation. Int J Mol Sci. 10.3390/ijms242316941 38069264 10.3390/ijms242316941 PMC10707166

[CR7] Cheng R, Gong L, Li Z, Liang YK (2019) Rice BIG gene is required for seedling viability. J Plant Physiol 232:39–50. 10.1016/j.jplph.2018.11.006 30530202 10.1016/j.jplph.2018.11.006

[CR8] Dai Z, Wang J, Yang X, Lu H, Miao X, Shi Z (2018) Modulation of plant architecture by the miR156f–OsSPL7–OsGH3.8 pathway in rice. J Exper Botany 69(21):5117–5130. 10.1093/jxb/ery273 30053063 10.1093/jxb/ery273 PMC6184515

[CR9] Dedow LK, Oren E, Braybrook SA (2022) Fake news blues: A GUS staining protocol to reduce false-negative data. Plant Direct 6(2):e367. 10.1002/pld3.367 35198848 10.1002/pld3.367 PMC8842172

[CR10] Dellaporta SL, Wood J, Hicks JB (1983) A plant DNA minipreparation: Version II. Plant Mol Biol Rep 1(4):19–21. 10.1007/BF02712670

[CR11] Ding G, Li Z, Iqbal Z, Zhao M, Cui Z, Cao L, Zhou J, Lei L, Luo Y, Bai L, Yang G, Wang R, Li K, Wang X, Liu K, Qu M, Sun S (2025) Identifications of genes involved in ABA and MAPK signaling pathways positively regulating cold tolerance in rice. Plants 14(4):498 40006757 10.3390/plants14040498 PMC11859393

[CR12] Figueiredo DD, Barros PM, Cordeiro AM, Serra TS, Lourenço T, Chander S, Oliveira MM, Saibo NJ (2012) Seven zinc-finger transcription factors are novel regulators of the stress responsive gene OsDREB1B. J Exp Bot 63(10):3643–3656. 10.1093/jxb/ers035 22412187 10.1093/jxb/ers035

[CR13] Fu C, Yang XO, Chen X, Chen W, Ma Y, Hu J, Li S (2009) OsEF3, a homologous gene of *Arabidopsis* ELF3, has pleiotropic effects in rice. Plant Biol (Stuttg) 11(5):751–757. 10.1111/j.1438-8677.2008.00156.x 19689783 10.1111/j.1438-8677.2008.00156.x

[CR14] Fukagawa NK, Ziska LH (2019) Rice: importance for global nutrition. J Nutr Sci Vitaminol (Tokyo) 65(Supplement):S2-s3. 10.3177/jnsv.65.S2 31619630 10.3177/jnsv.65.S2

[CR15] Guo J, Wang H, Guan W, Guo Q, Wang J, Yang J, Peng Y, Shan J, Gao M, Shi S, Shangguan X, Liu B, Jing S, Zhang J, Xu C, Huang J, Rao W, Zheng X, Wu D, He G (2023) A tripartite rheostat controls self-regulated host plant resistance to insects. Nature 618(7966):799–807. 10.1038/s41586-023-06197-z 37316670 10.1038/s41586-023-06197-z PMC10284691

[CR16] Guo M, Zheng C, Shi C, Lu X, She Z, Jiang S, Tian D, Qin Y (2025) The OsZHD1 and OsZHD2, two zinc finger homeobox transcription factor, redundantly control grain size by influencing cell proliferation in rice. Rice N Y 18(1):20. 10.1186/s12284-025-00774-8 40119214 10.1186/s12284-025-00774-8 PMC11928714

[CR17] Han M, Jin X, Yao W, Kong L, Huang G, Tao Y, Li L, Wang X, Wang Y (2017) A mini zinc-finger protein (MIF) from *Gerbera hybrida* activates the GASA protein family gene, GEG, to inhibit ray petal elongation. Front Plant Sci. 10.3389/fpls.2017.01649. (**[Original Research]**) 29018462 10.3389/fpls.2017.01649 PMC5615213

[CR18] Hong SY, Kim OK, Kim SG, Yang MS, Park CM (2011) Nuclear import and DNA binding of the ZHD5 transcription factor is modulated by a competitive peptide inhibitor in *Arabidopsis*. J Biol Chem 286(2):1659–1668. 10.1074/jbc.M110.167692 21059647 10.1074/jbc.M110.167692 PMC3020774

[CR19] Hori K, Sun J (2022) Rice grain size and quality. Rice N Y 15(1):33. 10.1186/s12284-022-00579-z 35776387 10.1186/s12284-022-00579-z PMC9249952

[CR20] Hu W, Ma H (2006) Characterization of a novel putative zinc finger gene MIF1: involvement in multiple hormonal regulation of Arabidopsis development. Plant J 45(3):399–422. 10.1111/j.1365-313X.2005.02626.x 16412086 10.1111/j.1365-313X.2005.02626.x

[CR21] Hu W, DePamphilis CW, Ma H (2008) Phylogenetic analysis of the plant-specific zinc finger-homeobox and mini zinc finger gene families. J Integr Plant Biol 50(8):1031–1045. 10.1111/j.1744-7909.2008.00681.x 18713354 10.1111/j.1744-7909.2008.00681.x

[CR22] Hu W, Feng B, Ma H (2011) Ectopic expression of the *Arabidopsis* MINI ZINC FINGER1 and MIF3 genes induces shoot meristems on leaf margins. Plant Mol Biol 76(1):57–68. 10.1007/s11103-011-9768-y 21455630 10.1007/s11103-011-9768-y

[CR23] Hu J, Huang L, Chen G, Liu H, Zhang Y, Zhang R, Zhang S, Liu J, Hu Q, Hu F, Wang W, Ding Y (2021) The elite alleles of OsSPL4 regulate grain size and increase grain yield in rice. Rice N Y 14(1):90. 10.1186/s12284-021-00531-7 34727228 10.1186/s12284-021-00531-7 PMC8563897

[CR24] Islam MAU, Nupur JA, Khalid MHB, Din AMU, Shafiq M, Alshegaihi RM, Ali Q, Ali Q, Kamran Z, Manzoor M, Haider MS, Shahid MA, Manghwar H (2022) Genome-wide identification and in silico analysis of ZF-HD transcription factor genes in *Zea mays* L. Genes Basel 13(11):2112 36421787 10.3390/genes13112112 PMC9690586

[CR25] Jain M, Nijhawan A, Tyagi AK, Khurana JP (2006) Validation of housekeeping genes as internal control for studying gene expression in rice by quantitative real-time PCR. Biochem Biophys Res Commun 345(2):646–651. 10.1016/j.bbrc.2006.04.140 16690022 10.1016/j.bbrc.2006.04.140

[CR26] Jain M, Tyagi AK, Khurana JP (2008) Genome-wide identification, classification, evolutionary expansion and expression analyses of homeobox genes in rice. FEBS J 275(11):2845–2861. 10.1111/j.1742-4658.2008.06424.x 18430022 10.1111/j.1742-4658.2008.06424.x

[CR27] Jan A, Maruyama K, Todaka D, Kidokoro S, Abo M, Yoshimura E, Shinozaki K, Nakashima K, Yamaguchi-Shinozaki K (2013) OsTZF1, a CCCH-tandem zinc finger protein, confers delayed senescence and stress tolerance in rice by regulating stress-related genes. Plant Physiol 161(3):1202–1216. 10.1104/pp.112.205385 23296688 10.1104/pp.112.205385 PMC3585590

[CR28] Jang MJ, Hong WJ, Park YS, Jung KH, Kim S (2023) Genomic basis of multiphase evolution driving divergent selection of zinc-finger homeodomain genes. Nucleic Acids Res 51(14):7424–7437. 10.1093/nar/gkad489 37394281 10.1093/nar/gkad489 PMC10415114

[CR29] Kim Y-M, Lee J-Y, Lee T, Lee Y-H, Kim S-H, Kang S-H, Yoon U-H, Ha S-H, Lim S-H (2012) The suppression of the glutelin storage protein gene in transgenic rice seeds results in a higher yield of recombinant protein. Plant Biotechnol Rep 6(4):347–353. 10.1007/s11816-012-0230-7

[CR30] Kim J-B, Kang J-Y, Park MY, Song M-R, Kim YC, Kim SY (2019) Arabidopsis zinc finger homeodomain protein ZHD5 promotes shoot regeneration and confers other cytokinin-related phenotypes when overexpressed. Plant Cell Tissue Organ Cult (PCTOC) 137(1):181–185. 10.1007/s11240-018-01546-7

[CR31] Kumar S, Kumar S, Krishnan GS, Mohapatra T (2022) Molecular basis of genetic plasticity to varying environmental conditions on growing rice by dry/direct-sowing and exposure to drought stress: insights for DSR varietal development. Front Plant Sci 13:1013207. 10.3389/fpls.2022.1013207 36352870 10.3389/fpls.2022.1013207 PMC9638133

[CR32] Kurusu T, Hamada J, Nokajima H, Kitagawa Y, Kiyoduka M, Takahashi A, Hanamata S, Ohno R, Hayashi T, Okada K, Koga J, Hirochika H, Yamane H, Kuchitsu K (2010) Regulation of microbe-associated molecular pattern-induced hypersensitive cell death, phytoalexin production, and defense gene expression by calcineurin B-like protein-interacting protein kinases, OsCIPK14/15, in rice cultured cells. Plant Physiol 153(2):678–692. 10.1104/pp.109.151852 20357140 10.1104/pp.109.151852 PMC2879771

[CR33] Lee Y, Kende H (2002) Expression of alpha-expansin and expansin-like genes in deepwater rice. Plant Physiol 130(3):1396–1405. 10.1104/pp.008888 12428004 10.1104/pp.008888 PMC166658

[CR34] Lescot M, Déhais P, Thijs G, Marchal K, Moreau Y, Van de Peer Y, Rouzé P, Rombauts S (2002) PlantCARE, a database of plant cis-acting regulatory elements and a portal to tools for in silico analysis of promoter sequences. Nucleic Acids Res 30(1):325–327. 10.1093/nar/30.1.325 11752327 10.1093/nar/30.1.325 PMC99092

[CR35] Li Z, Trick HN (2005) Rapid method for high-quality RNA isolation from seed endosperm containing high levels of starch. Biotechniques 38(6):872–874. 10.2144/05386BM05 16018547 10.2144/05386BM05

[CR36] Li P, Li H, Liu Z, Zhuang Y, Wei M, Gu Y, Liu Y, Sun X, Tang Y, Yue L, Lu L, Luo D, Huang W, Tu S, Wang S (2020) Characterization of the ‘Oat-Like Rice’ caused by a novel allele OsMADS1Olr reveals vital importance of OsMADS1 in regulating grain shape in *Oryza sativa* L. Rice 13(1):73. 10.1186/s12284-020-00428-x 33063229 10.1186/s12284-020-00428-x PMC7561663

[CR37] Li G, Tang J, Zheng J, Chu C (2021a) Exploration of rice yield potential: Decoding agronomic and physiological traits. Crop J 9(3):577–589. 10.1016/j.cj.2021.03.014

[CR38] Li G, Zhang H, Li J, Zhang Z, Li Z (2021b) Genetic control of panicle architecture in rice. The Crop Journal 9(3):590–597. 10.1016/j.cj.2021.02.004

[CR39] Li P, Chen Y-H, Lu J, Zhang C-Q, Liu Q-Q, Li Q-F (2022) Genes and their molecular functions determining seed structure, components, and quality of rice. Rice 15(1):18. 10.1186/s12284-022-00562-8 35303197 10.1186/s12284-022-00562-8 PMC8933604

[CR40] Li T, Li B, Wang Y, Xu J, Li W, Chen Z-H, Mou W, Xue D (2025) WRKY transcription factors in rice: key regulators orchestrating development and stress resilience. Plant Cell Environ. 10.1111/pce.70124 40831341 10.1111/pce.70124

[CR41] Lian L, Xu H, Zhang H, He W, Cai Q, Lin Y, Wei L, Pan L, Xie X, Zheng Y, Wei Y, Zhu Y, Xie H, Zhang J (2020) Overexpression of OsSPL14 results in transcriptome and physiology changes in indica rice ‘MH86.’ Plant Growth Regul 90(2):265–278. 10.1007/s10725-019-00569-0

[CR42] Livak KJ, Schmittgen TD (2001) Analysis of relative gene expression data using real-time quantitative PCR and the 2(-Delta Delta C(T)) method. Methods 25(4):402–408. 10.1006/meth.2001.1262 11846609 10.1006/meth.2001.1262

[CR43] Love MI, Huber W, Anders S (2014) Moderated estimation of fold change and dispersion for RNA-seq data with DESeq2. Genome Biol 15(12):550. 10.1186/s13059-014-0550-8 25516281 10.1186/s13059-014-0550-8 PMC4302049

[CR44] McCarthy DJ, Chen Y, Smyth GK (2012) Differential expression analysis of multifactor RNA-Seq experiments with respect to biological variation. Nucleic Acids Res 40(10):4288–4297. 10.1093/nar/gks042 22287627 10.1093/nar/gks042 PMC3378882

[CR45] Mishra N, Sun L, Zhu X, Smith J, Prakash Srivastava A, Yang X, Pehlivan N, Esmaeili N, Luo H, Shen G, Jones D, Auld D, Burke J, Payton P, Zhang H (2017) Overexpression of the rice SUMO E3 ligase gene OsSIZ1 in cotton enhances drought and heat tolerance, and substantially improves fiber yields in the field under reduced irrigation and rainfed conditions. Plant Cell Physiol 58(4):735–746. 10.1093/pcp/pcx032 28340002 10.1093/pcp/pcx032 PMC5444567

[CR46] Mishra N, Srivastava AP, Esmaeili N, Hu W, Shen G (2018) Overexpression of the rice gene OsSIZ1 in *Arabidopsis* improves drought-, heat-, and salt-tolerance simultaneously. PLoS ONE 13(8):e0201716. 10.1371/journal.pone.0201716 30092010 10.1371/journal.pone.0201716 PMC6084956

[CR47] Mohidem NA, Hashim N, Shamsudin R, Che Man H (2022) Rice for food security: Revisiting its production, diversity, rice milling process and nutrient content. Agriculture 12(6):741

[CR48] Niu H, Xia P, Hu Y, Zhan C, Li Y, Gong S, Li Y, Ma D (2021) Genome-wide identification of ZF-HD gene family in *Triticum aestivum*: molecular evolution mechanism and function analysis. PLoS ONE 16(9):e0256579. 10.1371/journal.pone.0256579 34559835 10.1371/journal.pone.0256579 PMC8462724

[CR49] Park HC, Kim H, Koo SC, Park HJ, Cheong MS, Hong H, Baek D, Chung WS, Kim DH, Bressan RA, Lee SY, Bohnert HJ, Yun DJ (2010) Functional characterization of the SIZ/PIAS-type SUMO E3 ligases, OsSIZ1 and OsSIZ2 in rice. Plant Cell Environ 33(11):1923–1934. 10.1111/j.1365-3040.2010.02195.x 20561251 10.1111/j.1365-3040.2010.02195.x

[CR50] Pathan AK, Bond J, Gaskin RE (2008) Sample preparation for scanning electron microscopy of plant surfaces--horses for courses. Micron 39(8):1049–1061. 10.1016/j.micron.2008.05.006 18586502 10.1016/j.micron.2008.05.006

[CR51] Pathan AK, Bond J, Gaskin RE (2010) Sample preparation for SEM of plant surfaces. Mater Today 12:32–43. 10.1016/S1369-7021(10)70143-7

[CR52] Perrella G, Davidson MLH, O’Donnell L, Nastase AM, Herzyk P, Breton G, Pruneda-Paz JL, Kay SA, Chory J, Kaiserli E (2018) Zinc-finger interactions mediate transcriptional regulation of hypocotyl growth in *Arabidopsis*. Proc Natl Acad Sci U S A 115(19):E4503-e4511. 10.1073/pnas.1718099115 29686058 10.1073/pnas.1718099115 PMC5948964

[CR53] Pham HA, Cho K, Tran AD, Chandra D, So J, Nguyen HTT, Sang H, Lee JY, Han O (2024) Compensatory modulation of seed storage protein synthesis and alteration of starch accumulation by selective editing of 13 kDa prolamin genes by CRISPR-Cas9 in rice. Int J Mol Sci. 10.3390/ijms25126579 38928285 10.3390/ijms25126579 PMC11204006

[CR54] Ren D, Ding C, Qian Q (2023) Molecular bases of rice grain size and quality for optimized productivity. Sci Bull 68(3):314–350. 10.1016/j.scib.2023.01.026 10.1016/j.scib.2023.01.026 36710151

[CR55] Sarkar NK, Thapar U, Kundnani P, Panwar P, Grover A (2013) Functional relevance of J-protein family of rice (Oryza sativa). Cell Stress Chaperones 18(3):321–331. 10.1007/s12192-012-0384-9 23160806 10.1007/s12192-012-0384-9 PMC3631087

[CR56] Segami S, Takehara K, Yamamoto T, Kido S, Kondo S, Iwasaki Y, Miura K (2017) Overexpression of SRS5 improves grain size of brassinosteroid-related dwarf mutants in rice (*Oryza sativa* L.). Breed Sci 67(4):393–397. 10.1270/jsbbs.16198 29085249 10.1270/jsbbs.16198 PMC5654457

[CR57] Shalmani A, Muhammad I, Sharif R, Zhao C, Ullah U, Zhang D, Jing XQ, Amin B, Jia P, Mobeen Tahir M, Xu Z, Chen KM, An N (2019) Zinc finger-homeodomain genes: evolution, functional differentiation, and expression profiling under flowering-related treatments and abiotic stresses in plants. Evol Bioinform Online 15:1176934319867930. 10.1177/1176934319867930 31523124 10.1177/1176934319867930 PMC6728664

[CR58] Shen A-Q, Lv M-Y, Ge Y-X, Zhou J, Hu Z-Z, Ren X-Q, Xiong A-S, Wang G-L (2025) Zinc finger-homeodomain transcription factor: a new player in plant growth, stress response, and quality regulation. Agronomy 15(7):1522

[CR59] Shim Y, Lim C, Seong G, Choi Y, Kang K, Paek N-C (2022) The AP2/ERF transcription factor LATE FLOWERING SEMI-DWARF suppresses long-day-dependent repression of flowering. Plant Cell Environ 45(8):2446–2459. 10.1111/pce.14365 35610056 10.1111/pce.14365

[CR60] Shin JH, Jeong DH, Park MC, An G (2005) Characterization and transcriptional expression of the alpha-expansin gene family in rice. Mol Cells 20(2):210–218 16267395

[CR61] Si L, Chen J, Huang X, Gong H, Luo J, Hou Q, Zhou T, Lu T, Zhu J, Shangguan Y, Chen E, Gong C, Zhao Q, Jing Y, Zhao Y, Li Y, Cui L, Fan D, Lu Y, Han B (2016) OsSPL13 controls grain size in cultivated rice. Nat Genet 48(4):447–456. 10.1038/ng.3518 26950093 10.1038/ng.3518

[CR62] So J, Lee J-Y, Cho K, Park S, Lee K, Kim D-K, Han O (2025) Transcriptome-based identification of novel transcription factors regulating seed storage proteins in rice. Plants 14(17):2791 40941954 10.3390/plants14172791 PMC12431501

[CR63] Thangasamy S, Guo C-L, Chuang M-H, Lai M-H, Chen J, Jauh G-Y (2011) Rice SIZ1, a SUMO E3 ligase, controls spikelet fertility through regulation of anther dehiscence. New Phytol 189(3):869–882. 10.1111/j.1469-8137.2010.03538.x 21083564 10.1111/j.1469-8137.2010.03538.x

[CR64] Thiaw M, Meunier A, Dardou A, Roques S, Jouannic S, Tran H, Guyomarc'H S, Masram G, Lazennec F, Moukouanga D, Colin M, Vernet A, Portefaix M, Saoud J, Affortit P, Brossier J-R, Tregear J, Doridant I, Sallaud C, Gantet P (2025) Ectopic expression of the OsMIF1 microprotein alters rice plant development and promotes drought tolerance. 10.2139/ssrn.5934630

[CR65] Thiaw MRN, Gantet P (2024) The emerging functions of mini zinc finger (MIF) microproteins in seed plants: a minireview. Biochimie 218:69–75. 10.1016/j.biochi.2023.09.016 37722501 10.1016/j.biochi.2023.09.016

[CR66] Thiaw MRN (2024) Caractérisation fonctionnelle de gènes impliqués dans le développement des racines coronaires chez le riz (Publication Number s408943) [Doctoral thesis, Université de Montpellier]. Montpellier, France. https://theses.fr/s408943

[CR67] Tran LS, Nakashima K, Sakuma Y, Osakabe Y, Qin F, Simpson SD, Maruyama K, Fujita Y, Shinozaki K, Yamaguchi-Shinozaki K (2007) Co-expression of the stress-inducible zinc finger homeodomain ZFHD1 and NAC transcription factors enhances expression of the ERD1 gene in Arabidopsis. Plant J 49(1):46–63. 10.1111/j.1365-313X.2006.02932.x 17233795 10.1111/j.1365-313X.2006.02932.x

[CR68] Wang H, Makeen K, Yan Y, Cao Y, Sun S, Xu G (2011a) OsSIZ1 regulates the vegetative growth and reproductive development in rice. Plant Mol Biol Rep 29(2):411–417. 10.1007/s11105-010-0232-y

[CR69] Wang L, Hua D, He J, Duan Y, Chen Z, Hong X, Gong Z (2011b) Auxin response factor2 (ARF2) and its regulated homeodomain gene HB33 mediate abscisic acid response in Arabidopsis. PLoS Genet 7(7):e1002172. 10.1371/journal.pgen.1002172 21779177 10.1371/journal.pgen.1002172 PMC3136439

[CR70] Wang H, Sun R, Cao Y, Pei W, Sun Y, Zhou H, Wu X, Zhang F, Luo L, Shen Q, Xu G, Sun S (2015a) OsSIZ1, a SUMO E3 ligase gene, is involved in the regulation of the responses to phosphate and nitrogen in rice. Plant Cell Physiol 56(12):2381–2395. 10.1093/pcp/pcv162 26615033 10.1093/pcp/pcv162

[CR71] Wang S, Li S, Liu Q, Wu K, Zhang J, Wang S, Wang Y, Chen X, Zhang Y, Gao C, Wang F, Huang H, Fu X (2015b) The OsSPL16-GW7 regulatory module determines grain shape and simultaneously improves rice yield and grain quality. Nat Genet 47(8):949–954. 10.1038/ng.3352 26147620 10.1038/ng.3352

[CR72] Wang X, He Y, Wei H, Wang L (2021) A clock regulatory module is required for salt tolerance and control of heading date in rice. Plant Cell Environ 44(10):3283–3301. 10.1111/pce.14167 34402093 10.1111/pce.14167

[CR73] Windhövel A, Hein I, Dabrowa R, Stockhaus J (2001) Characterization of a novel class of plant homeodomain proteins that bind to the C4 phosphoenolpyruvate carboxylase gene of *Flaveria trinervia*. Plant Mol Biol 45(2):201–214. 10.1023/A:1006450005648 11289511 10.1023/a:1006450005648

[CR74] Yang L, Fang S, Liu L, Zhao L, Chen W, Li X, Xu Z, Chen S, Wang H, Yu D (2025) WRKY transcription factors: hubs for regulating plant growth and stress responses. J Integr Plant Biol 67(3):488–509. 10.1111/jipb.13828 39815727 10.1111/jipb.13828

[CR75] Yoon J, Cho LH, Yang W, Pasriga R, Wu Y, Hong WJ, Bureau C, Wi SJ, Zhang T, Wang R, Zhang D, Jung KH, Park KY, Périn C, Zhao Y, An G (2020) Homeobox transcription factor OsZHD2 promotes root meristem activity in rice by inducing ethylene biosynthesis. J Exp Bot 71(18):5348–5364. 10.1093/jxb/eraa209 32449922 10.1093/jxb/eraa209 PMC7501826

[CR76] Yu J, Miao J, Zhang Z, Xiong H, Zhu X, Sun X, Pan Y, Liang Y, Zhang Q, Abdul Rehman RM, Li J, Zhang H, Li Z (2018) Alternative splicing of OsLG3b controls grain length and yield in japonica rice. Plant Biotechnol J 16(9):1667–1678. 10.1111/pbi.12903 29479793 10.1111/pbi.12903 PMC6097128

[CR77] Zhan P, Ma S, Xiao Z, Li F, Wei X, Lin S, Wang X, Ji Z, Fu Y, Pan J, Zhou M, Liu Y, Chang Z, Li L, Bu S, Liu Z, Zhu H, Liu G, Zhang G, Wang S (2022) Natural variations in grain length 10 (GL10) regulate rice grain size. J Genet Genomics 49(5):405–413. 10.1016/j.jgg.2022.01.008 35151907 10.1016/j.jgg.2022.01.008

[CR78] Zhang Y, Chen Z (2020) Broad and complex roles of NBR1-mediated selective autophagy in plant stress responses. Cells 9(12):2562 33266087 10.3390/cells9122562 PMC7760648

[CR79] Zhang Q, Shen L, Ren D, Hu J, Chen G, Zhu L, Gao Z, Zhang G, Guo L, Zeng D, Qian Q (2019) Characterization, expression, and interaction analyses of OsMORF gene family in rice. Genes. 10.3390/genes10090694 31509970 10.3390/genes10090694 PMC6770982

[CR80] Zhao J, Huang X, Ouyang X, Chen W, Du A, Zhu L, Wang S, Deng XW, Li S (2012) OsELF3-1, an ortholog of Arabidopsis early flowering 3, regulates rice circadian rhythm and photoperiodic flowering. PLoS ONE 7(8):e43705. 10.1371/journal.pone.0043705 22912900 10.1371/journal.pone.0043705 PMC3422346

[CR81] Zhao D, Zhang C, Li Q, Liu Q (2022) Genetic control of grain appearance quality in rice. Biotechnol Adv 60:108014. 10.1016/j.biotechadv.2022.108014 35777622 10.1016/j.biotechadv.2022.108014

[CR82] Zhong X, Yang J, Shi Y, Wang X, Wang GL (2018) The DnaJ protein OsDjA6 negatively regulates rice innate immunity to the blast fungus *Magnaporthe oryzae*. Mol Plant Pathol 19(3):607–614. 10.1111/mpp.12546 28220688 10.1111/mpp.12546 PMC6638105

[CR83] Zhou Y, Miao J, Gu H, Peng X, Leburu M, Yuan F, Gu H, Gao Y, Tao Y, Zhu J, Gong Z, Yi C, Gu M, Yang Z, Liang G (2015) Natural variations in SLG7 regulate grain shape in rice. Genetics 201(4):1591–1599. 10.1534/genetics.115.181115 26434724 10.1534/genetics.115.181115 PMC4676533

[CR84] Zuo ZW, Zhang ZH, Huang DR, Fan YY, Yu SB, Zhuang JY, Zhu YJ (2021) Control of thousand-grain weight by OsMADS56 in rice. Int J Mol Sci. 10.3390/ijms23010125 35008551 10.3390/ijms23010125 PMC8745348

