# Supplementary material for: Zinc Finger Homeobox Transcription Factors OsMIF1 and OsMIF2 Regulate Grain Size and Panicle Development in Rice
Source: Rice (N Y). 2026 Mar 10;19:28. doi: 10.1186/s12284-026-00898-5 (PMC13087070; doi:10.1186/s12284-026-00898-5)
Supplement: Supplementary file 1 — Supplementary Material 1. [file 12284_2026_898_MOESM1_ESM.zip › Supplementary Materials.docx]

**Supplementary Materials**

**Supplementary Fig. 1** Phylogenetic tree of rice ZF-HD family proteins constructed from the zinc finger motif sequences.

**Supplementary Fig. 2** Predicted three-dimensional structures of rice ZF-HD family proteins.

**Supplementary Fig. 3** Subcellular localization of OsMIF1 in rice protoplasts.

**Supplementary Fig. 4** Phylogenetic analysis of OsMIF1 orthologs collected from OrthoDB (https://www.orthodb.org/).

**Supplementary Fig. 5** Validation of the *pOsMIF1::GUS* transgenic rice lines.

**Supplementary Fig. 6** Histochemical staining in immature seeds of *pOsMIF1::GUS* transgenic rice plant.

**Supplementary Fig. 7** Prediction of cis-regulatory elements in the promoters of *OsMIF1* and *OsMIF2*.

**Supplementary Fig. 8** qRT-PCR analysis of *OsMIF1* and *OsMIF2* expression under hormone treatments.

**Supplementary Fig. 9** Validation of CRISPR-Cas9 mediated mutations in OsMIF1 and OsMIF2 knockout lines.

**Supplementary Fig. 10** Morphological traits of panicles in the NT and KO mutants.

**Supplementary Fig. 11** Transcriptome analysis of *osmif1*, *osmif2*, and *osmif1/osmif2* KO lines in immature seeds (14 DAF) based on RNA-seq.

**Supplementary Fig. 12** KEGG pathway-based classification of downregulated DEGs from RNA-seq of 10-cm developing panicles within the STRING interaction network.

**Supplementary Fig. 13** Correlation analysis between qRT-PCR and RNA-seq expression data in 10-cm developing panicles.

**Supplementary Fig. 14** Expression profiling of seed storage protein (SSP) genes of *osmif1*, *osmif2*, and *osmif1/osmif2* KO lines in immature seeds (14 DAF).

**Supplementary Fig. 15** Yeast two-hybrid (Y2H) library-scale screening.

**Supplementary Fig. 16** Gene Ontology (GO) enrichment analysis of proteins identified from the Y2H library-scale screening with OsMIF1.

**Supplementary Fig. 17** Germination rates of OsMIF1 and OsMIF2 KO mutant lines under salt stress.

**Supplementary Table 1** Primer list

**Supplementary Table 2** Information on ZF-HD family proteins

**Supplementary Table 3** Domain prediction of ZF-HD family proteins using InterProScan tool

**Supplementary Table 4** Genotype of OsMIF1 and OsMIF2 knock-out lines (T_0_ generation)

**Supplementary Table 5** Genotype of OsMIF1 and OsMIF2 knock-out lines (T_1_ generation)

**Supplementary Table 6** Genotype of OsMIF1 and OsMIF2 knock-out lines (T_2_ generation)

**Supplementary Table 7** RNA-seq statistics

**Supplementary Table 8** STRING-based KEGG pathway annotation of DEGs in 10-cm developing panicles of the mutants

**Supplementary Table 9** List of DEGs from KEGG pathways selected for RNA-seq heatmap and qRT-PCR validation

**Supplementary Table 10** List of photosynthesis-related genes selected for RNA-seq heatmap and qRT-PCR validation

**Supplementary Table 11** TMM-normalized counts of grain size and cell expansion related genes in 10-cm developing panicles of the mutants from RNA-seq analysis

**Supplementary Table 12** TMM-normalized counts of seed storage protein genes in 14 DAF immature seeds of the mutants from RNA-seq analysis

**Supplementary Table 13** Results of Yeast Two-Hybrid library-scale screening for OsMIF1

**Supplementary Table 14** Sequence analysis of candidate proteins interacting with OsMIF1

**Supplementary Table 15** GO analysis of in-frame candidate proteins interacting with OsMIF1


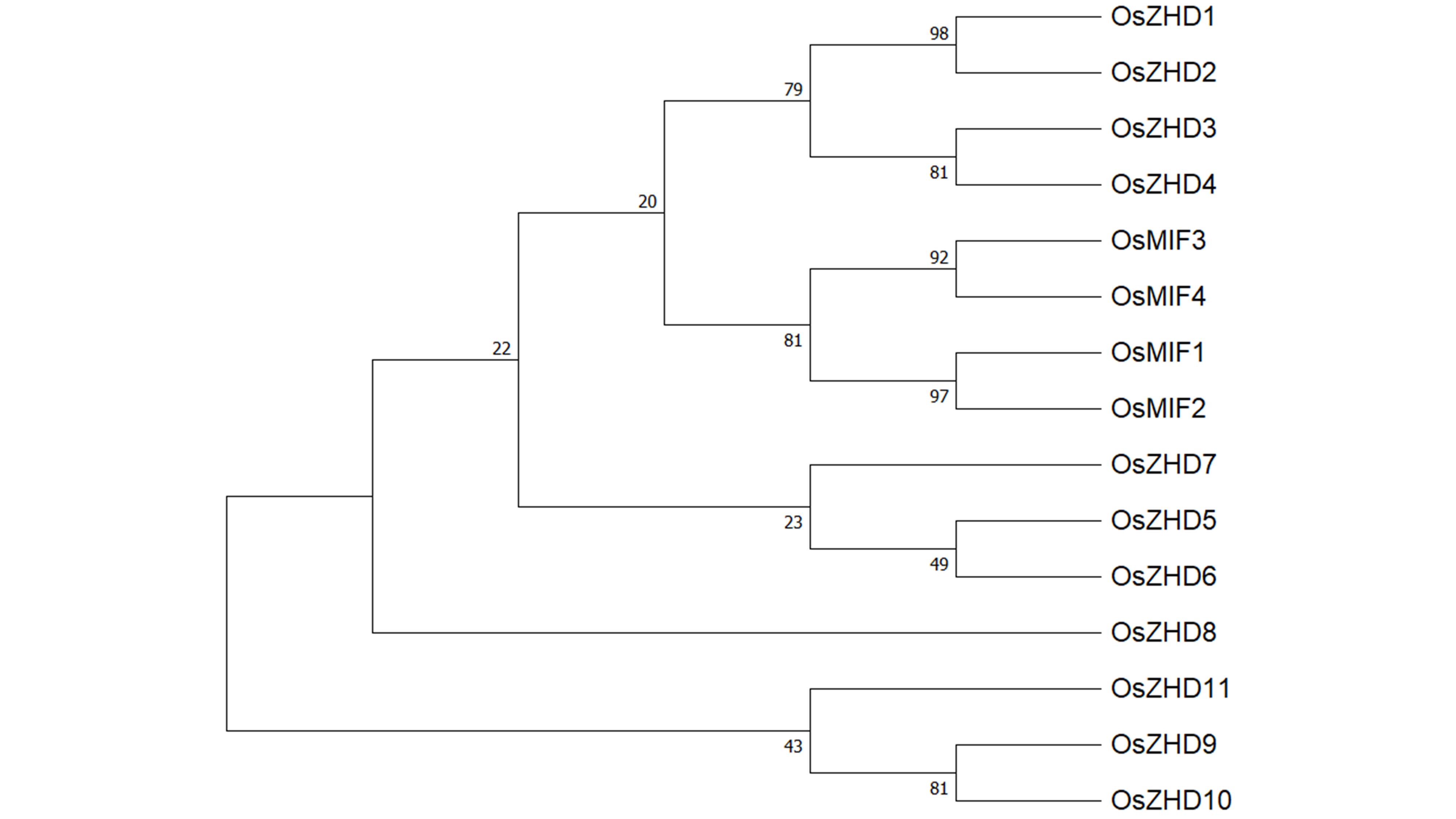


**Supplementary Fig. 1** Phylogenetic tree of rice ZF-HD family proteins constructed from the zinc finger motif sequences. The tree was constructed using the Maximum Likelihood method (1,000 bootstraps) in MEGA12, with bootstrap values shown at the branch nodes.


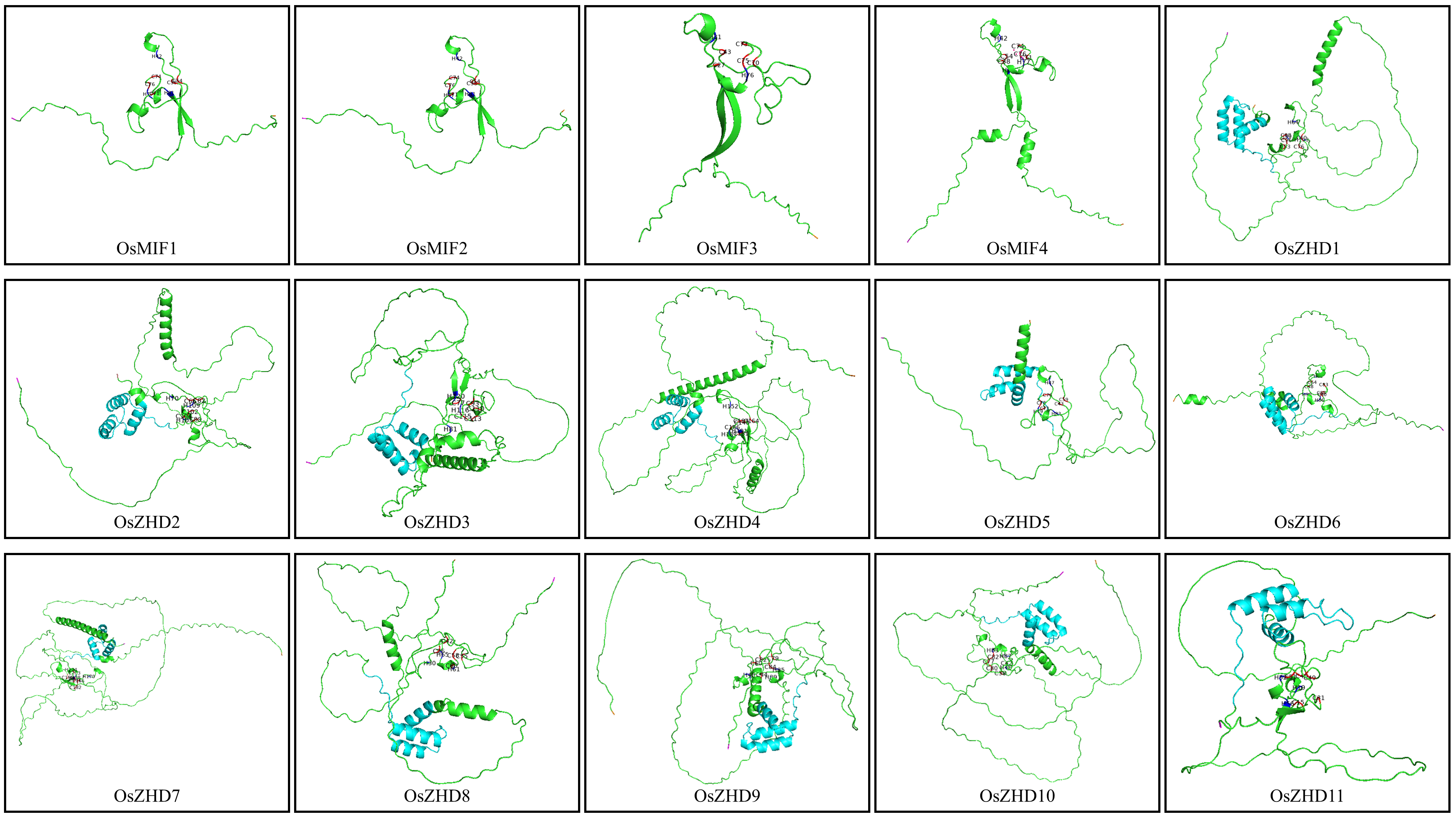


**Supplementary Fig. 2** Predicted three-dimensional structures of rice ZF-HD family proteins. Protein structures were predicted using AlphaFold (https://alphafold.ebi.ac.uk/).


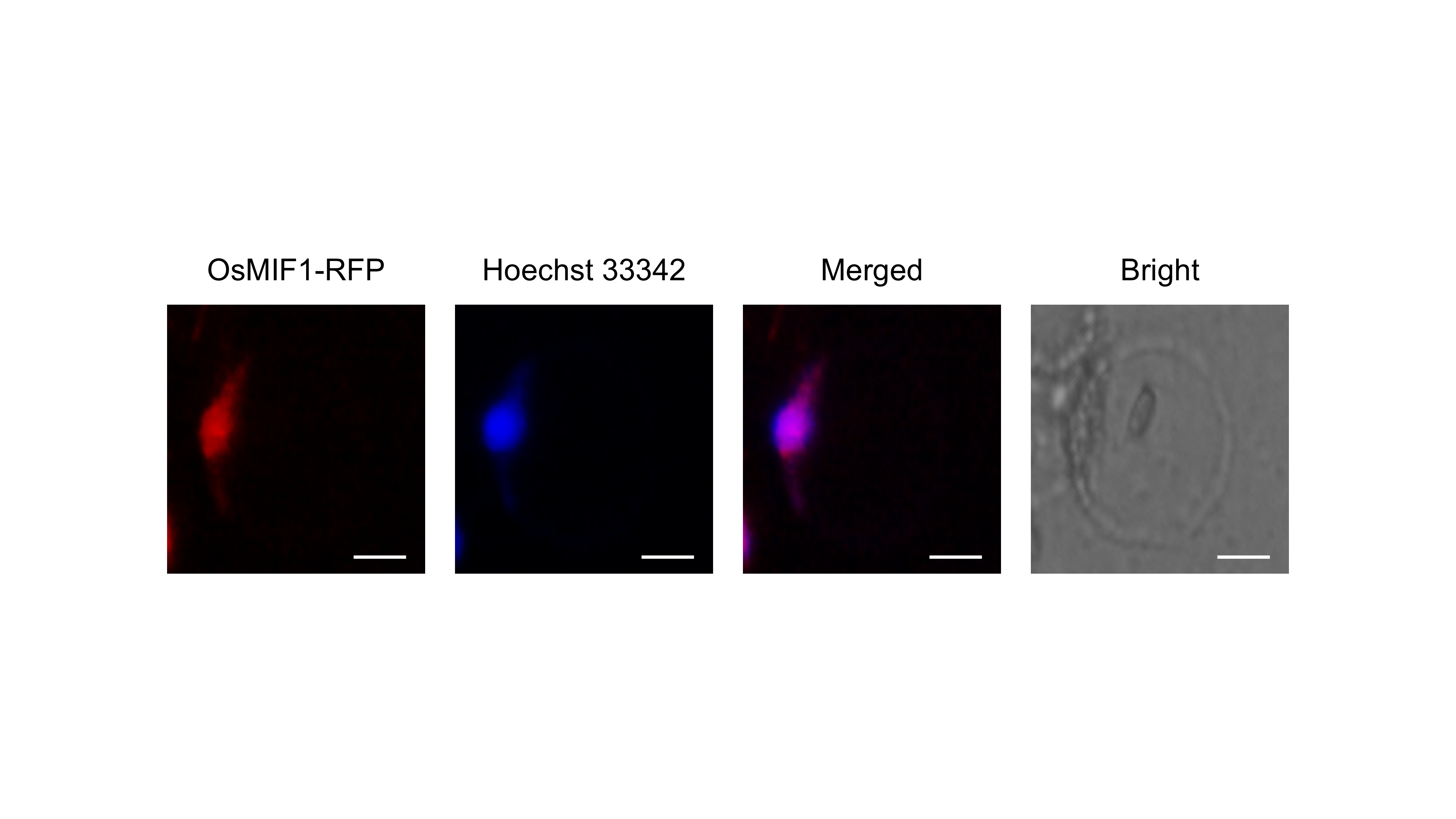


**Supplementary Fig. 3** Subcellular localization of OsMIF1 in rice protoplasts. OsMIF1–RFP was transiently expressed in rice protoplasts. Nuclei were stained with Hoechst 33342. Images were acquired using a confocal laser scanning microscope. Scale bars = 10 μm.


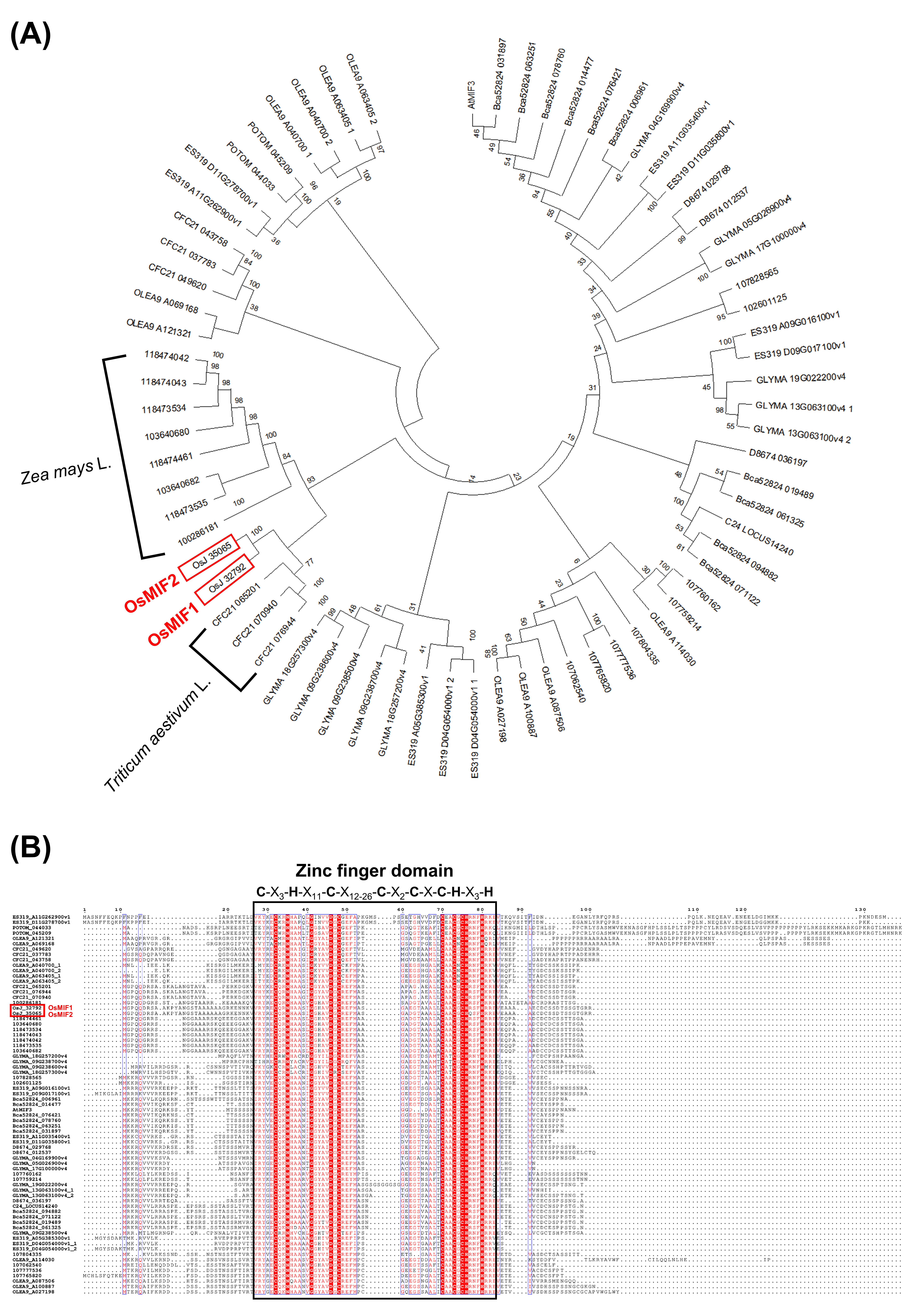


**Supplementary Fig. 4** Phylogenetic analysis of OsMIF1 orthologs collected from OrthoDB (https://www.orthodb.org/). **(A)** Phylogenetic tree of OsMIF1 orthologs using the Maximum Likelihood method (1,000 bootstraps) in MEGA12. Bootstrap values are shown at the branch nodes. OsMIF1 and OsMIF2 are indicated with red boxes. **(B)** Multiple sequence alignment of OsMIF1 and its orthologs from various species, highlighting the conserved zinc finger domain. The alignment was performed and visualized using ESPript (https://espript.ibcp.fr/ESPript/ESPript/).


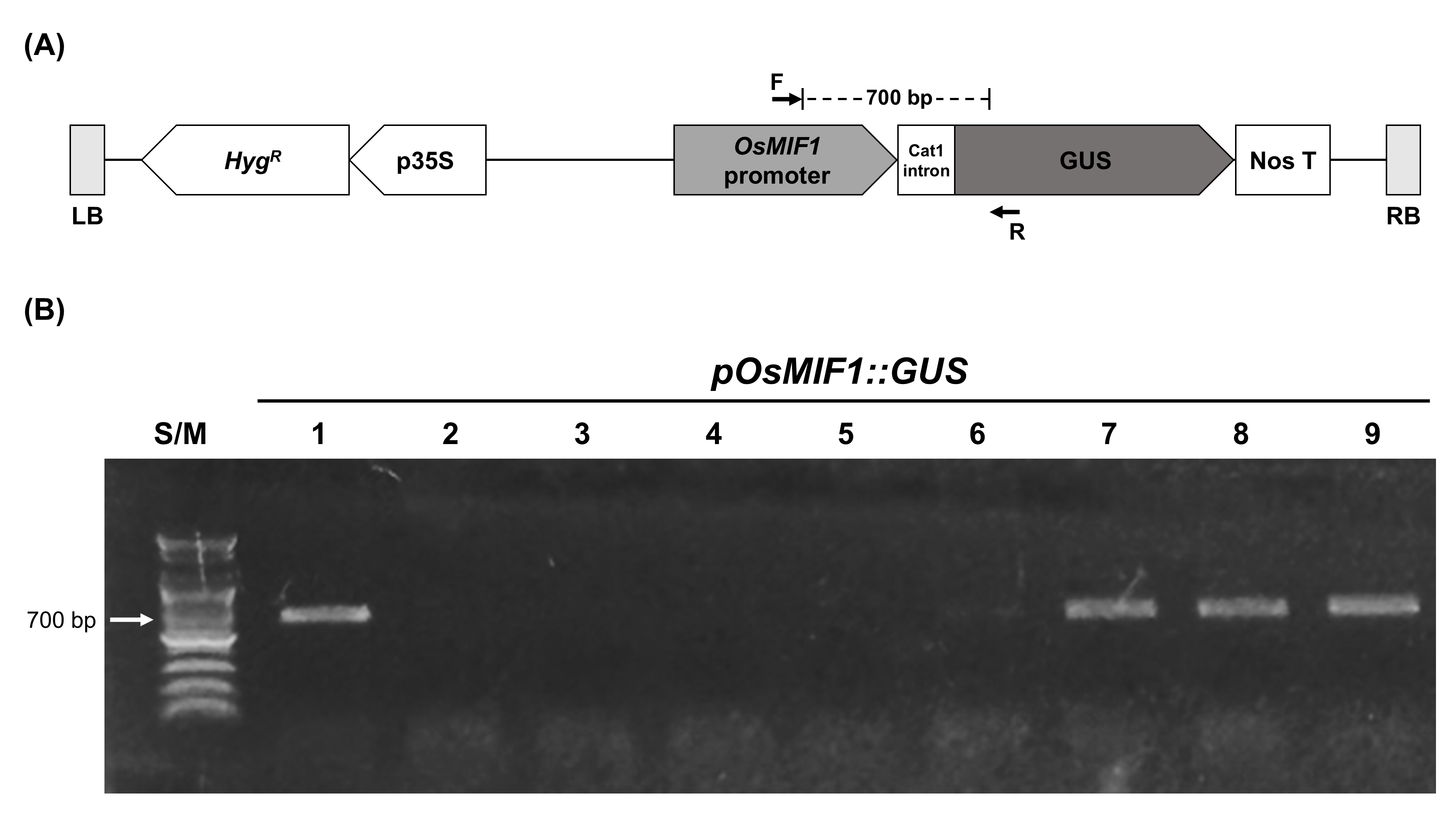


**Supplementary Fig. 5** Validation of the *pOsMIF1::GUS* transgenic rice lines. **(A)** Schematic diagram of *pOsMIF1::GUS* vector construction and primer positions used to detect the *pOsMIF1::GUS* insert. **(B)** Detection of the insert in transgenic rice by PCR analysis.


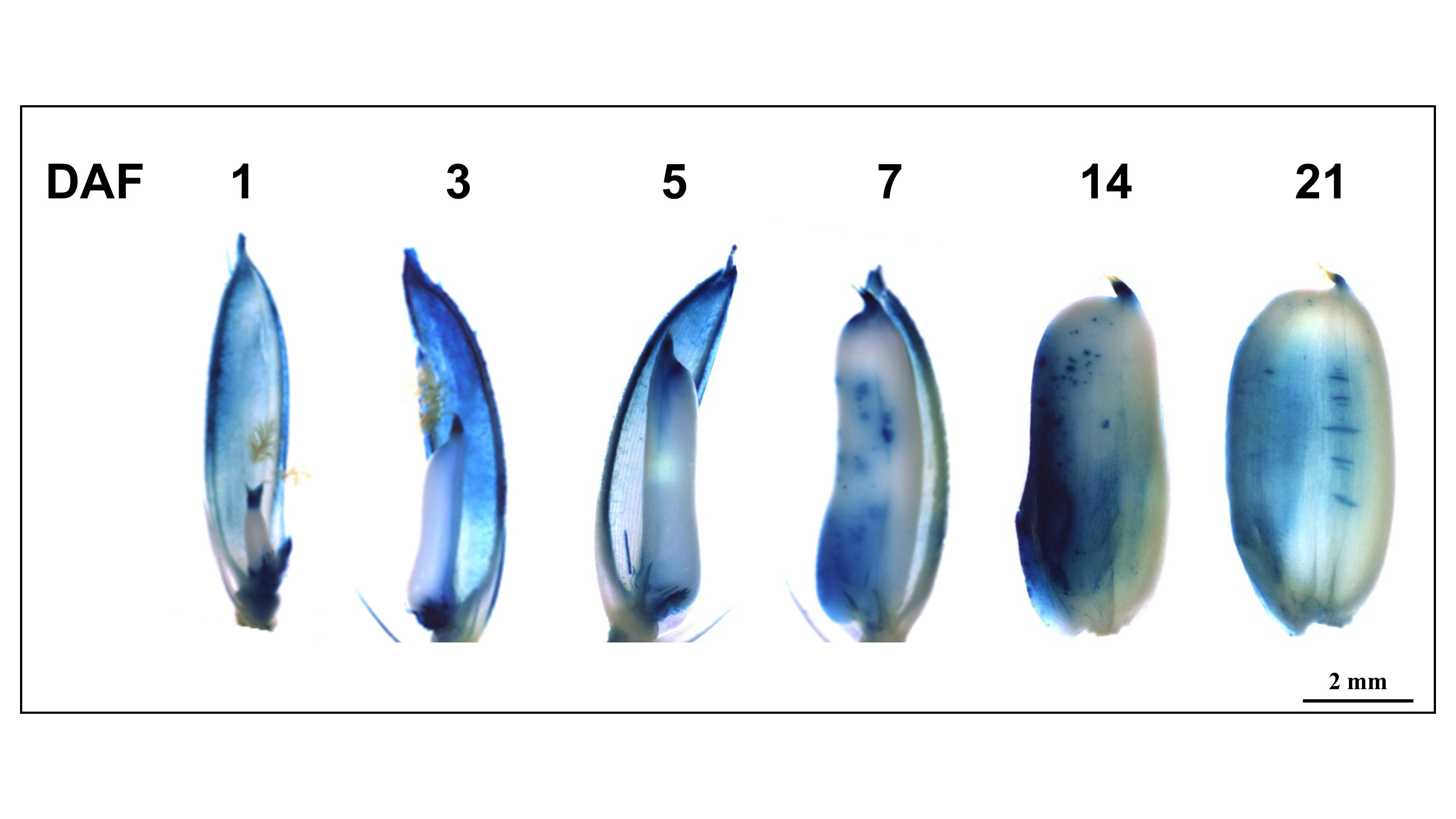


**Supplementary Fig. 6** Histochemical staining in immature seeds of *pOsMIF1::GUS* transgenic rice plant.


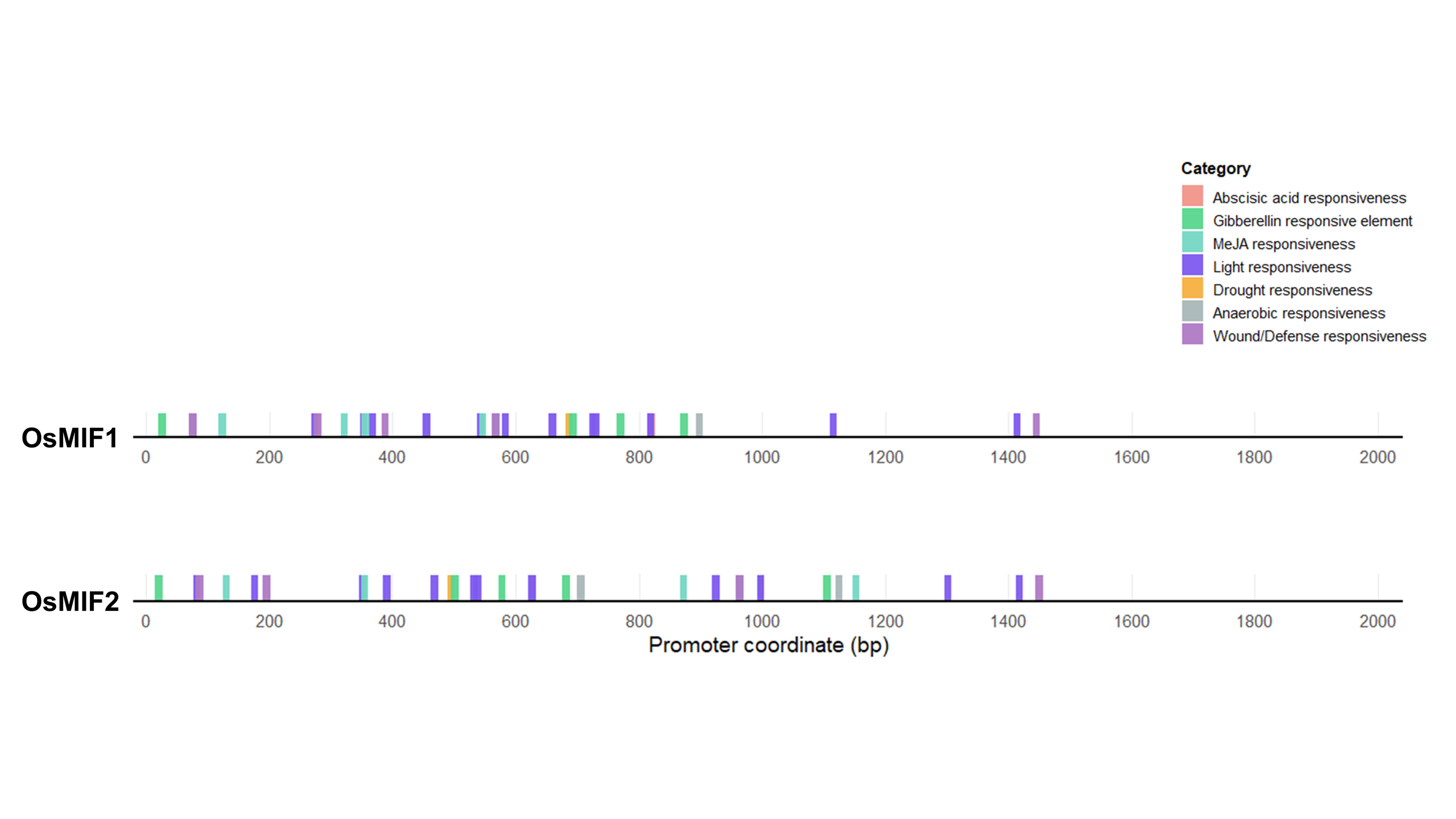


**Supplementary Fig. 7** Prediction of cis-regulatory elements in the promoters of *OsMIF1* and *OsMIF2*. Promoter regions (1.5-kb upstream of the start codon) of *OsMIF1* and *OsMIF2* were analyzed using the PlantCARE database (https://bioinformatics.psb.ugent.be/webtools/plantcare/html/).


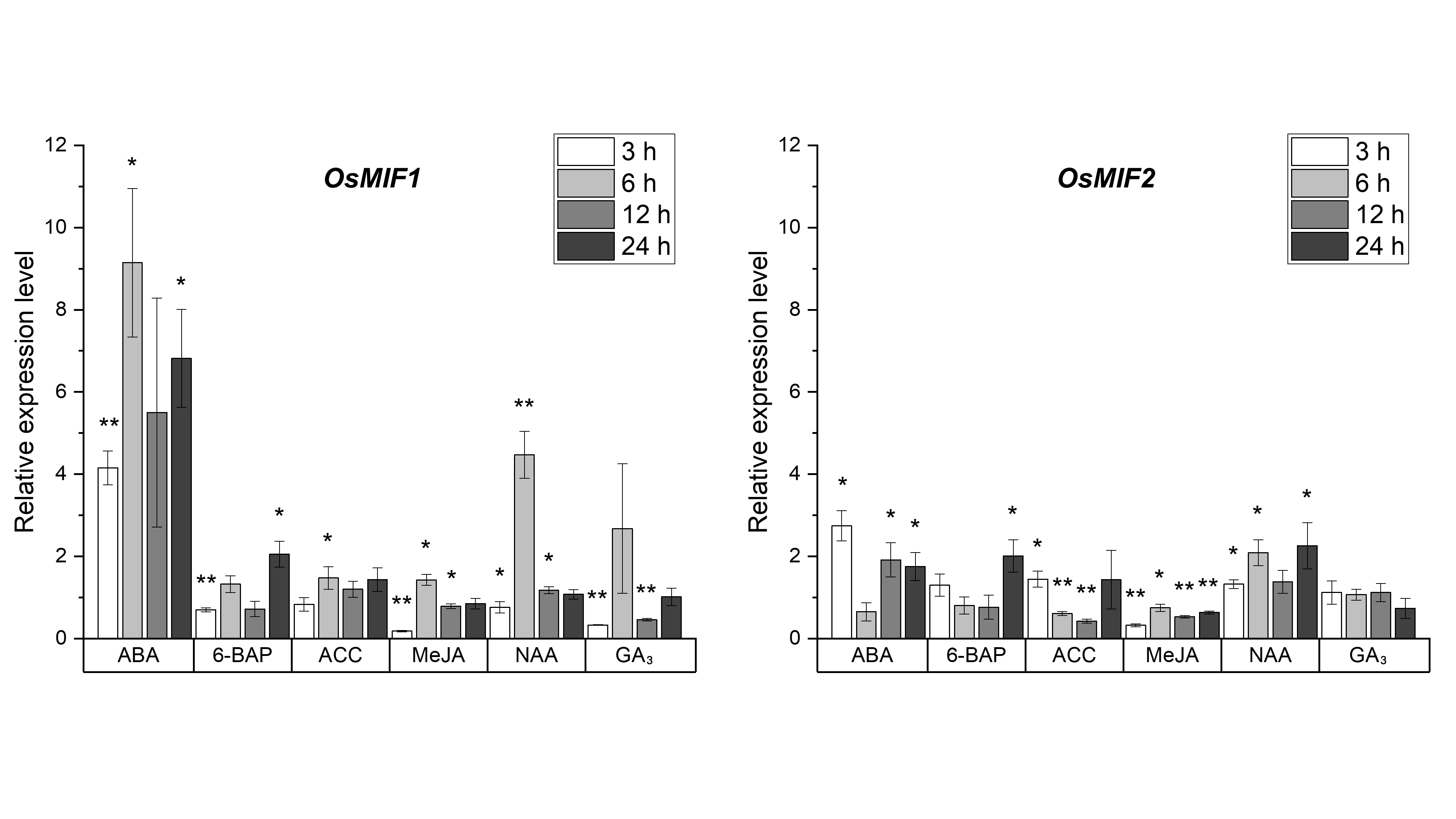


**Supplementary Fig. 8** qRT-PCR analysis of *OsMIF1* and *OsMIF2* expression under hormone treatments. Rice leaf discs were treated with ABA (200 μM), 6-BAP (100 μM), ACC (100 μM), MeJA (100 μM), NAA (1 mM), or GA_3_ (100 μM), and samples were collected at 3, 6, 12, and 24 h. Relative expression levels were calculated by normalizing to the corresponding non-treated control at each time point. Error bars represent SD (n = 3). *P*-values were calculated by the Student’s t-test (* p < 0.1, ** p < 0.01).


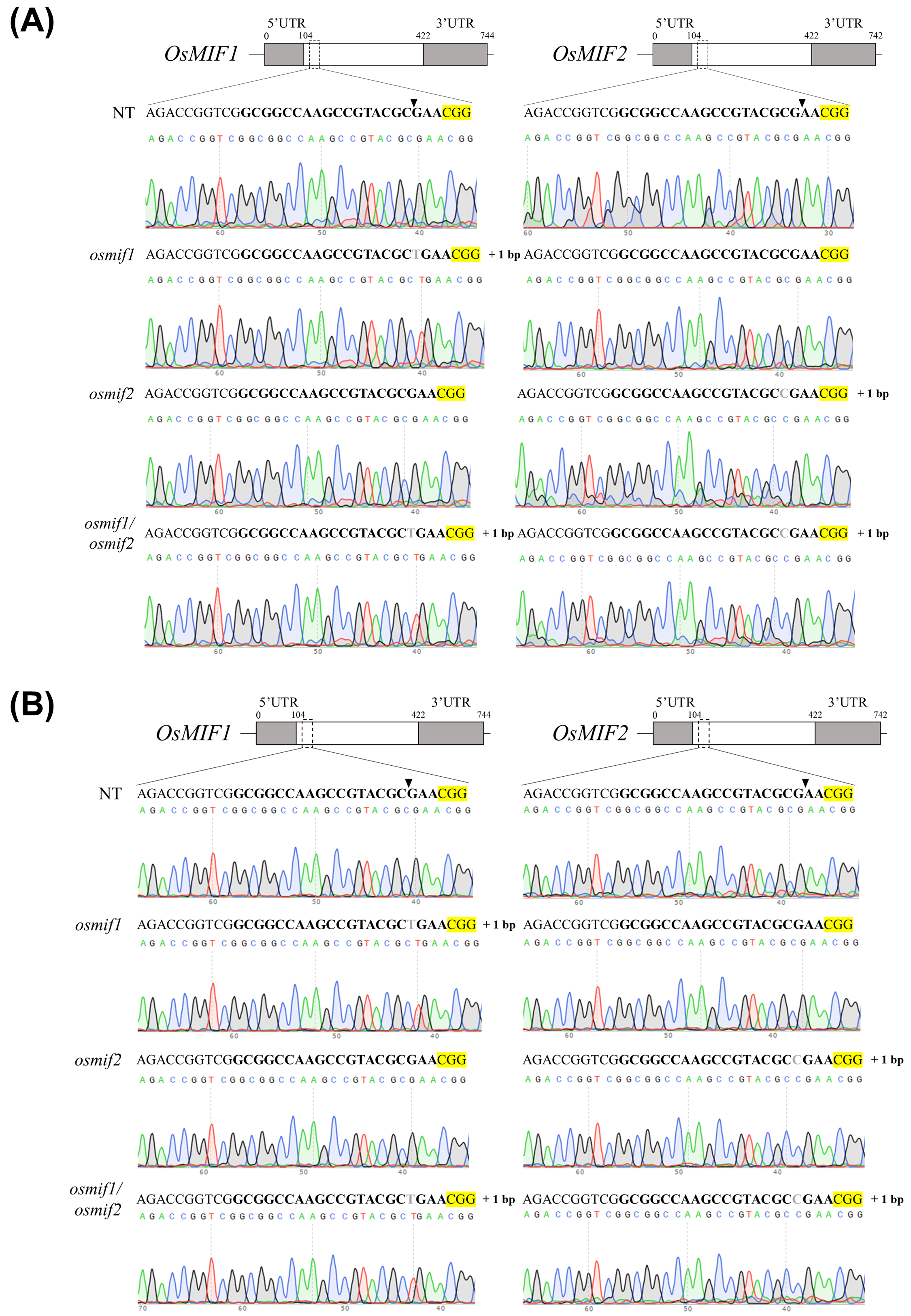

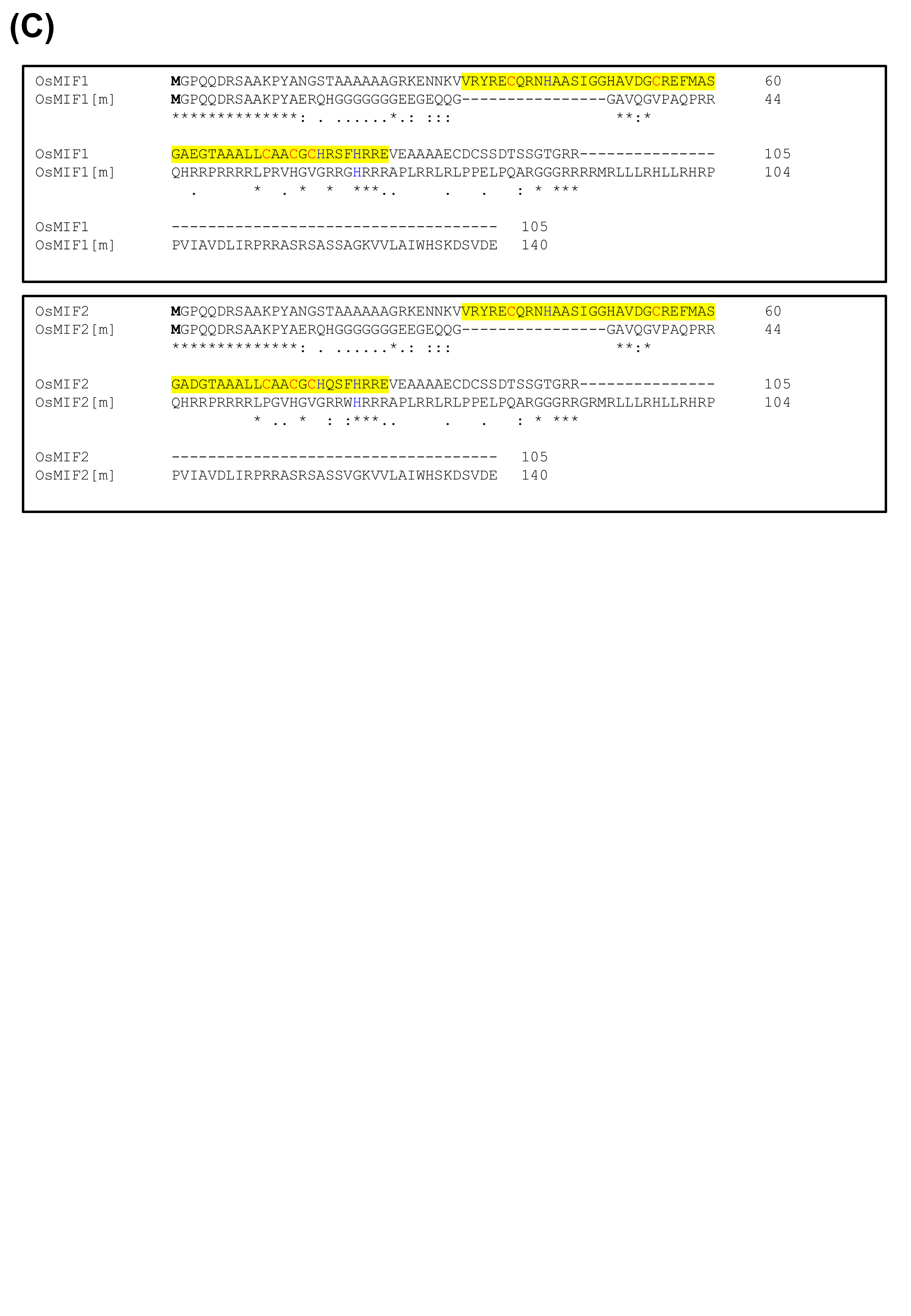


**Supplementary Fig. 9** Validation of CRISPR-Cas9 mediated mutations in OsMIF1 and OsMIF2 knockout lines. **(A)** Sanger sequencing chromatograms showing the CRISPR-Cas9 target sites of *OsMIF1* and *OsMIF2* in T_2_ knockout lines (genomic DNA). The target sequences and PAM motifs are highlighted in bold, and insertion sites are indicated. **(B)** Sanger sequencing chromatograms confirming the same mutation sites at the transcript level using cDNA synthesized from mRNA of the corresponding knockout lines. The target sequences and PAM motifs are highlighted in bold, and insertion sites are indicated. **(C)** Comparison of predicted amino acid sequences of OsMIF1 and OsMIF2 between wild-type and KO mutants. The conserved zinc finger domain is highlighted in yellow.


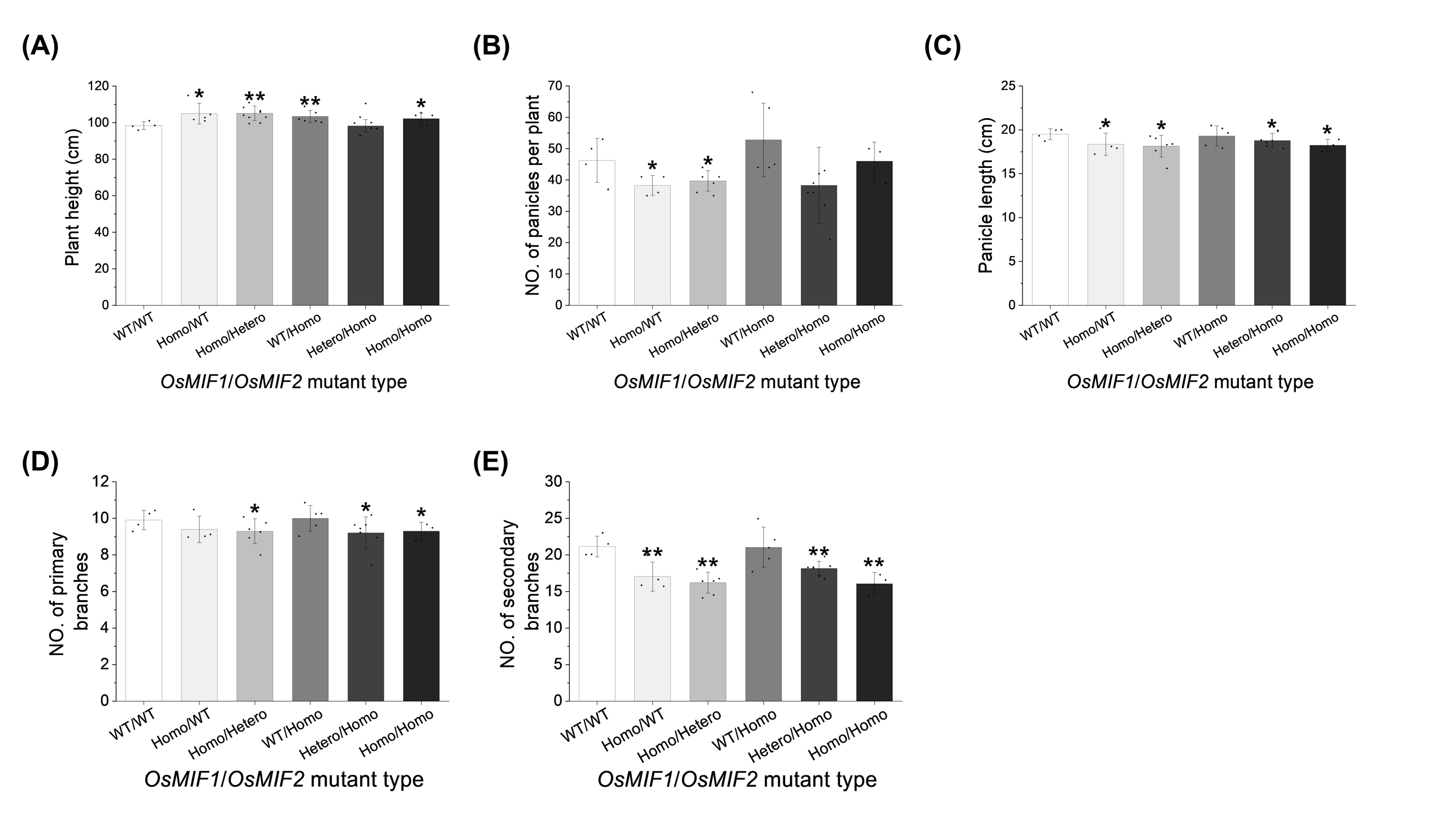


**Supplementary Fig. 10** Morphological traits of panicles in the NT and KO mutants. **(A)** Plant height, **(B)** Number of panicles per plant, **(C)** Panicle length, **(D)** Number of primary branches, and **(E)** Number of secondary branches. WT: wild type; Homo: homozygous; Hetero: heterozygous. Values are mean ± SD (3 ≤ n ≤ 10). *P*-values were calculated by the Student’s t-test (* p < 0.1, ** p < 0.01).


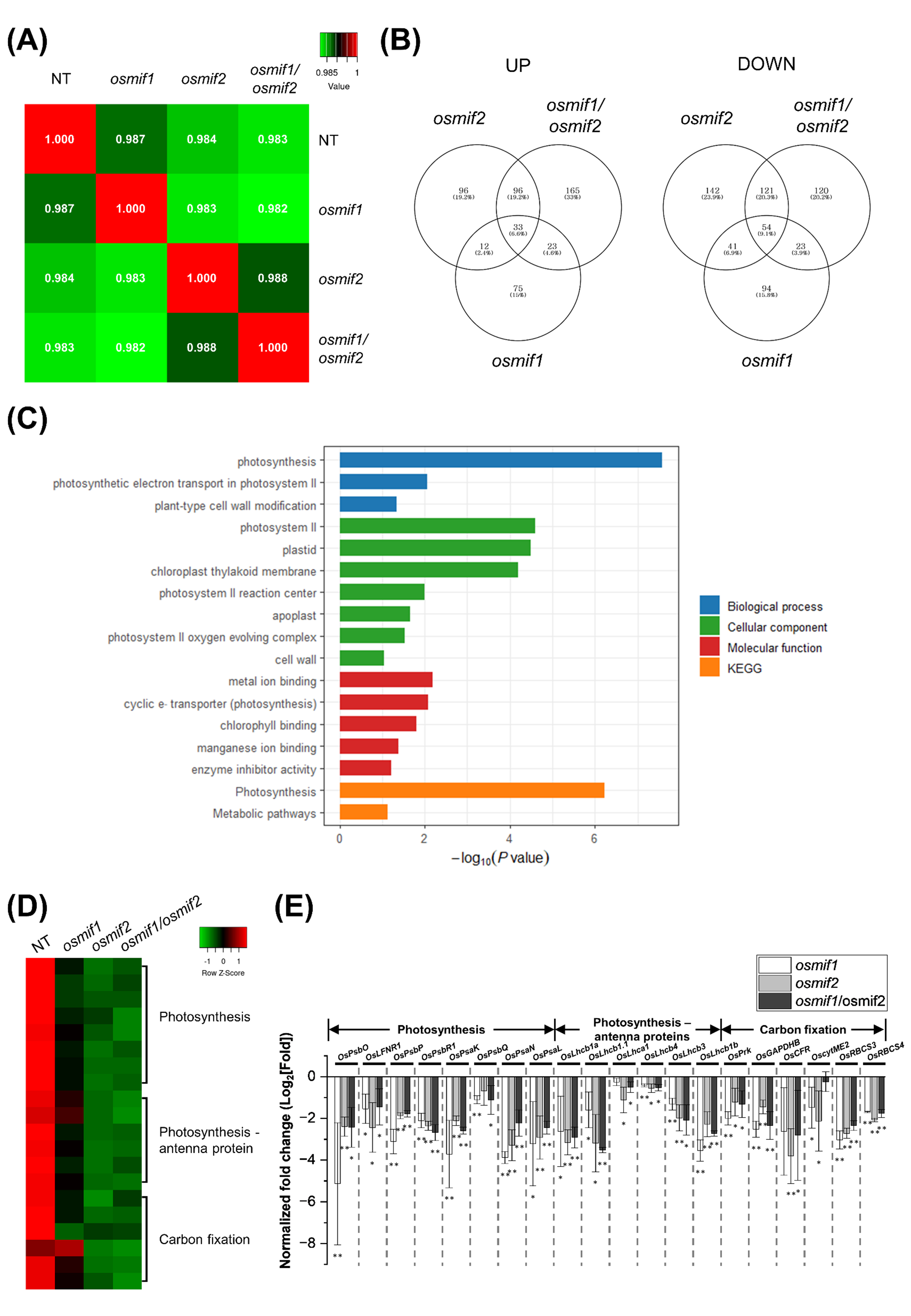


**Supplementary Fig. 11** Transcriptome analysis of *osmif1*, *osmif2*, and *osmif1/osmif2* KO lines in immature seeds (14 DAF) based on RNA-seq. **(A)** Pearson correlation matrix of transcriptomic profiles among NT, *osmif1*, *osmif2*, and *osmif1/osmif2* lines. **(B)** Venn diagrams of differentially expressed genes (DEGs) that are upregulated (left) or downregulated (right) in each KO line. **(C)** Gene ontology (GO) classification of downregulated DEGs using the DAVID tool. **(D-E)** Expression profiling of genes related to photosynthesis. **(D)** Heatmap based on RNA-seq data. Expression levels are shown as Z-score normalized values across samples. **(E)** qRT-PCR of genes related to photosynthesis. Normalization of expression levels of the target genes was performed using the 2^−ΔΔCT^ method and the relative level of individual gene in each OsMIF1 and OsMIF2 KO lines versus NT is represented as log_2_ (average fold change) value. *OsUBI5* was used as the internal control. Values are mean ± SD (n = 3). *P*-values were calculated using Student’s t-test (*p< 0.1, **p< 0.01).


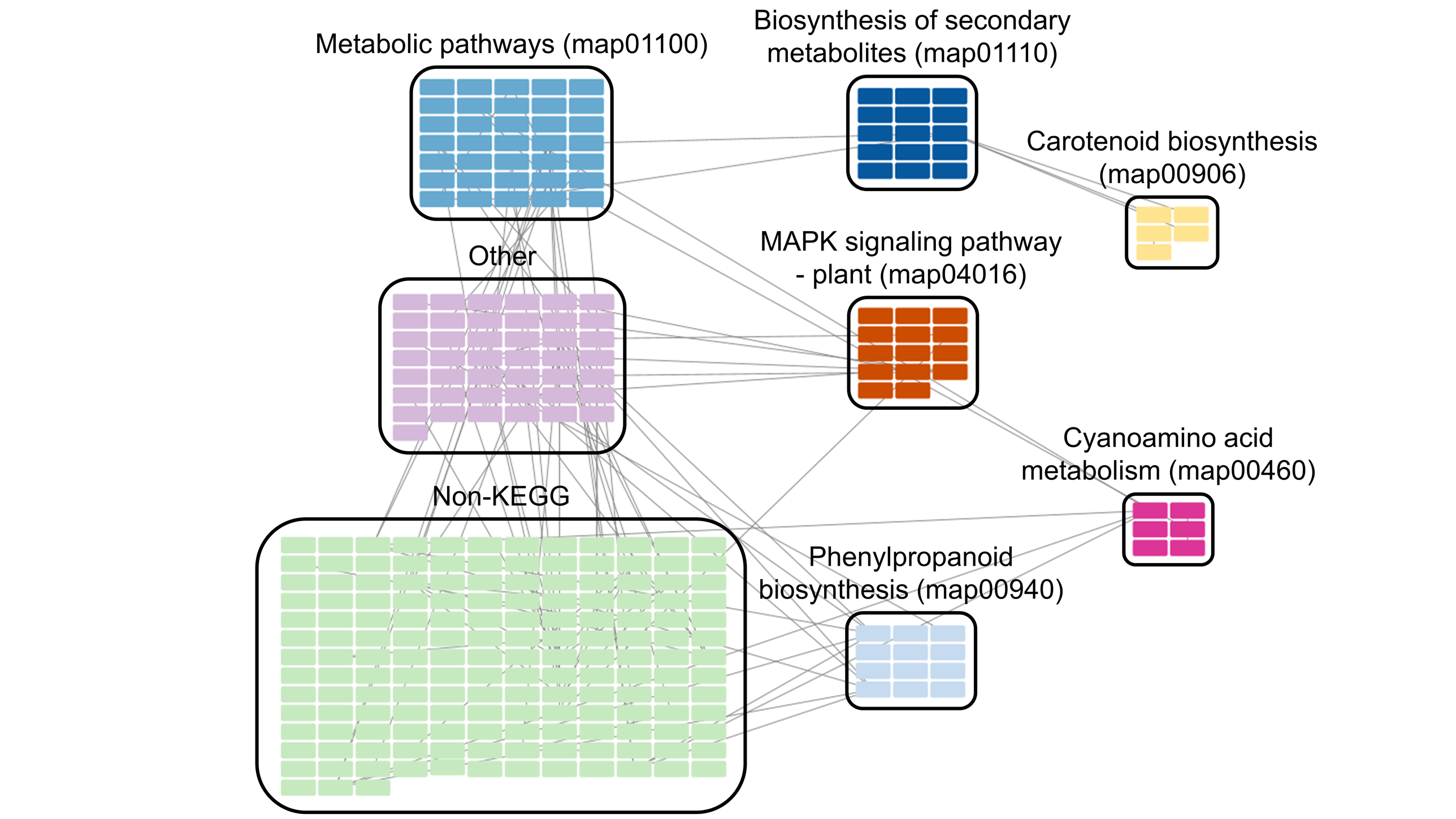


**Supplementary Fig. 12** KEGG pathway-based classification of downregulated DEGs from RNA-seq of 10-cm developing panicles within the STRING interaction network. Protein–protein interaction (PPI) network was constructed using the STRING database and visualized in Cytoscape. Each node represents a protein identified from the dataset, and edges indicate predicted or experimentally validated interactions.


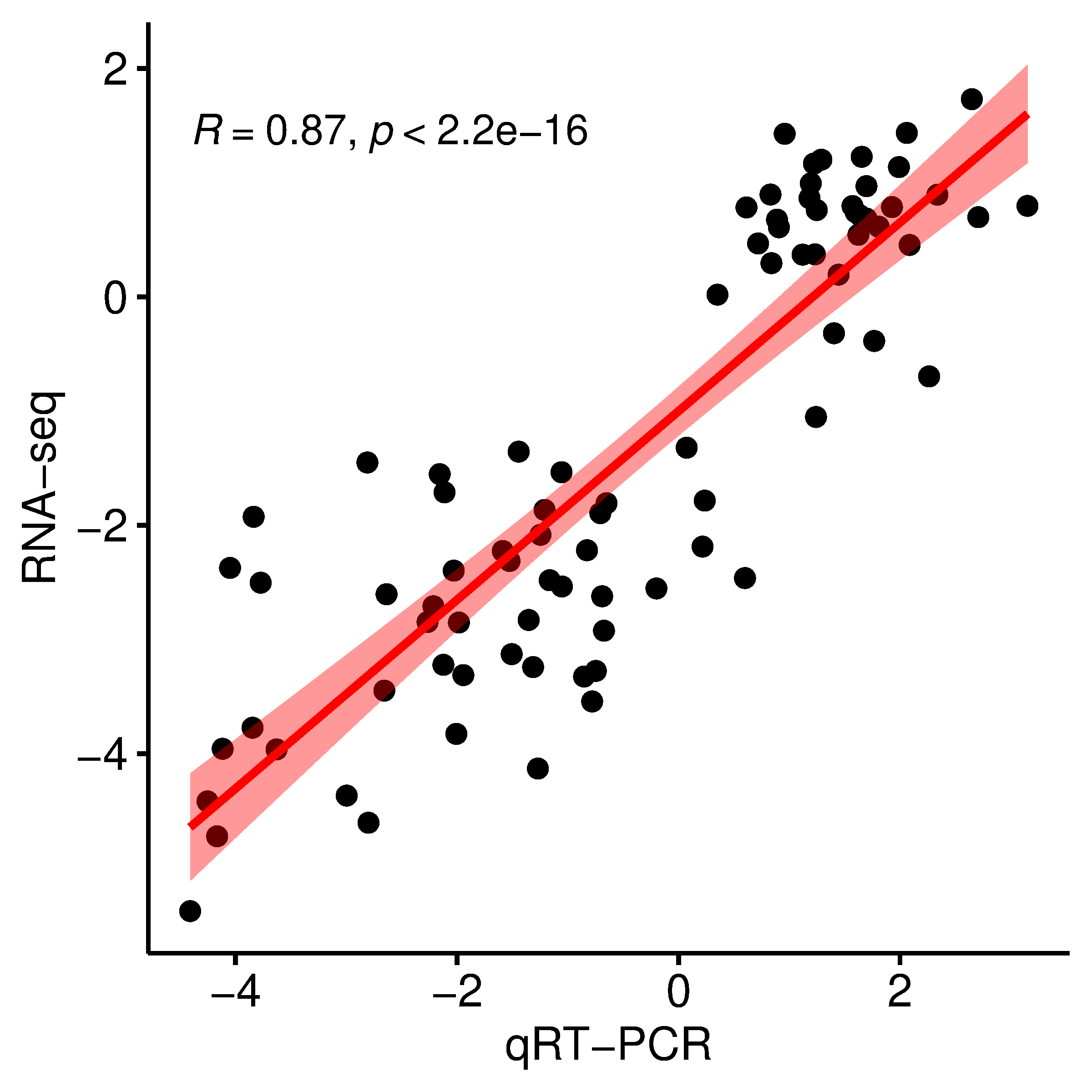


**Supplementary Fig. 13** Correlation analysis between qRT-PCR and RNA-seq expression data in 10-cm developing panicles. A regression scatter plot showing the relationship between relative gene expression levels measured by qRT-PCR (x-axis) and RNA-seq (y-axis). Regression analysis revealed a strong positive linear relationship between the two datasets (R² > 0.7). The qRT-PCR data were normalized using the 2^-ΔΔCT^ method and subsequently converted to log_2_ fold change (log_2_FC) values, and RNA-seq transcript data were presented as log_2_FC values. The plot was visualized using SRplot “Pearson spearman scatter” (https://www.bioinformatics.com.cn/plot_basic_pcc_scatter_plot_049_en).


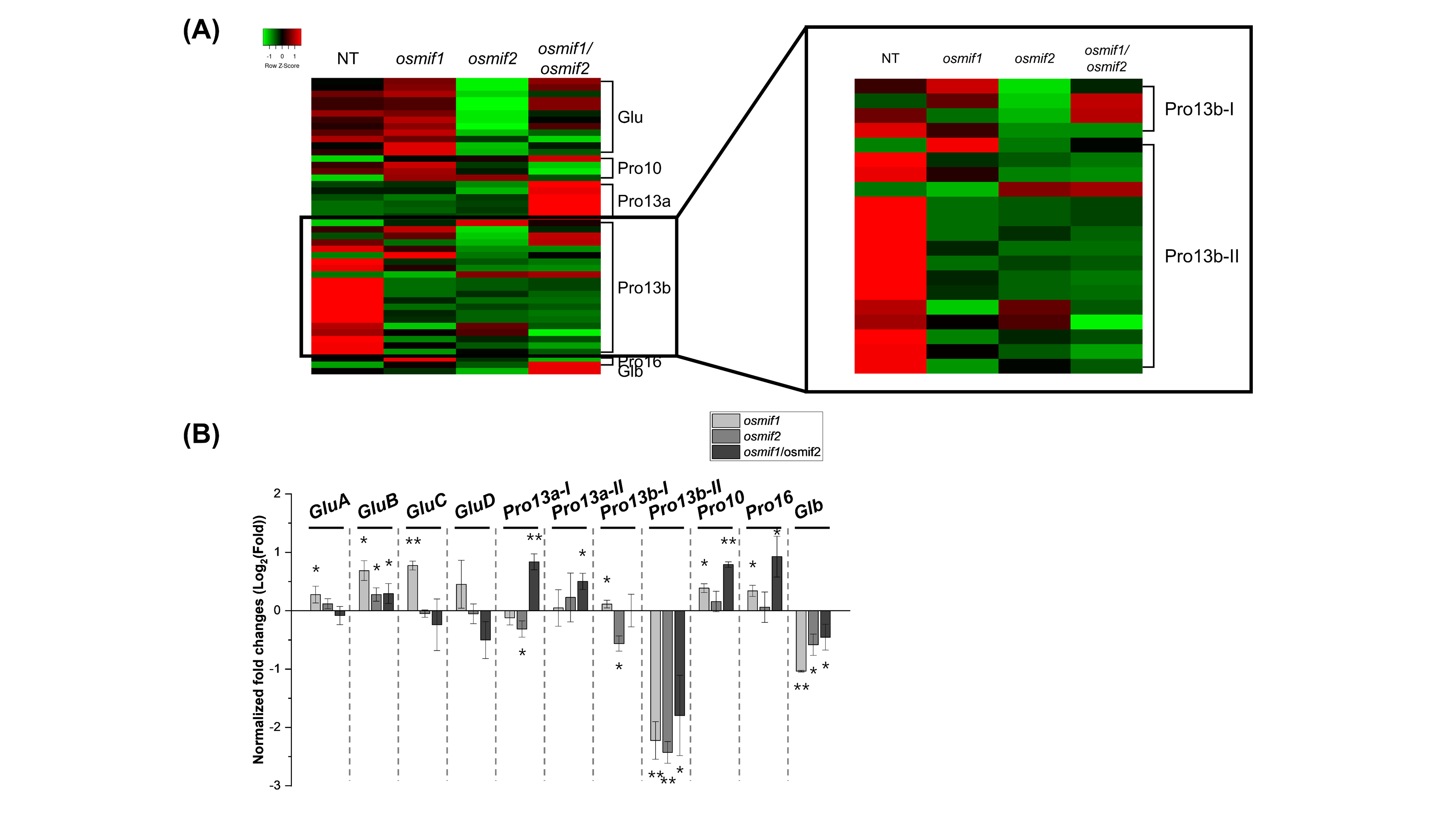


**Supplementary Fig. 14** Expression profiling of seed storage protein (SSP) genes of *osmif1*, *osmif2*, and *osmif1/osmif2* KO lines in immature seeds (14 DAF). **(A)** Heatmap based on RNA-seq data from immature seeds (14 DAF). Expression levels were calculated using TMM-normalized TPM values and are shown as Z-score normalized values across samples. **(B)** qRT-PCR of SSP genes. Normalization of expression levels of the target genes was performed using the 2^−ΔΔCT^ method and the relative level of each gene in the KO lines versus NT is presented as log_2_ (average fold change) value. *OsUBI5* was used as the internal control. Values are mean ± SD (n = 3). *P*-values were calculated using Student’s t-test (*p< 0.1, **p< 0.01).


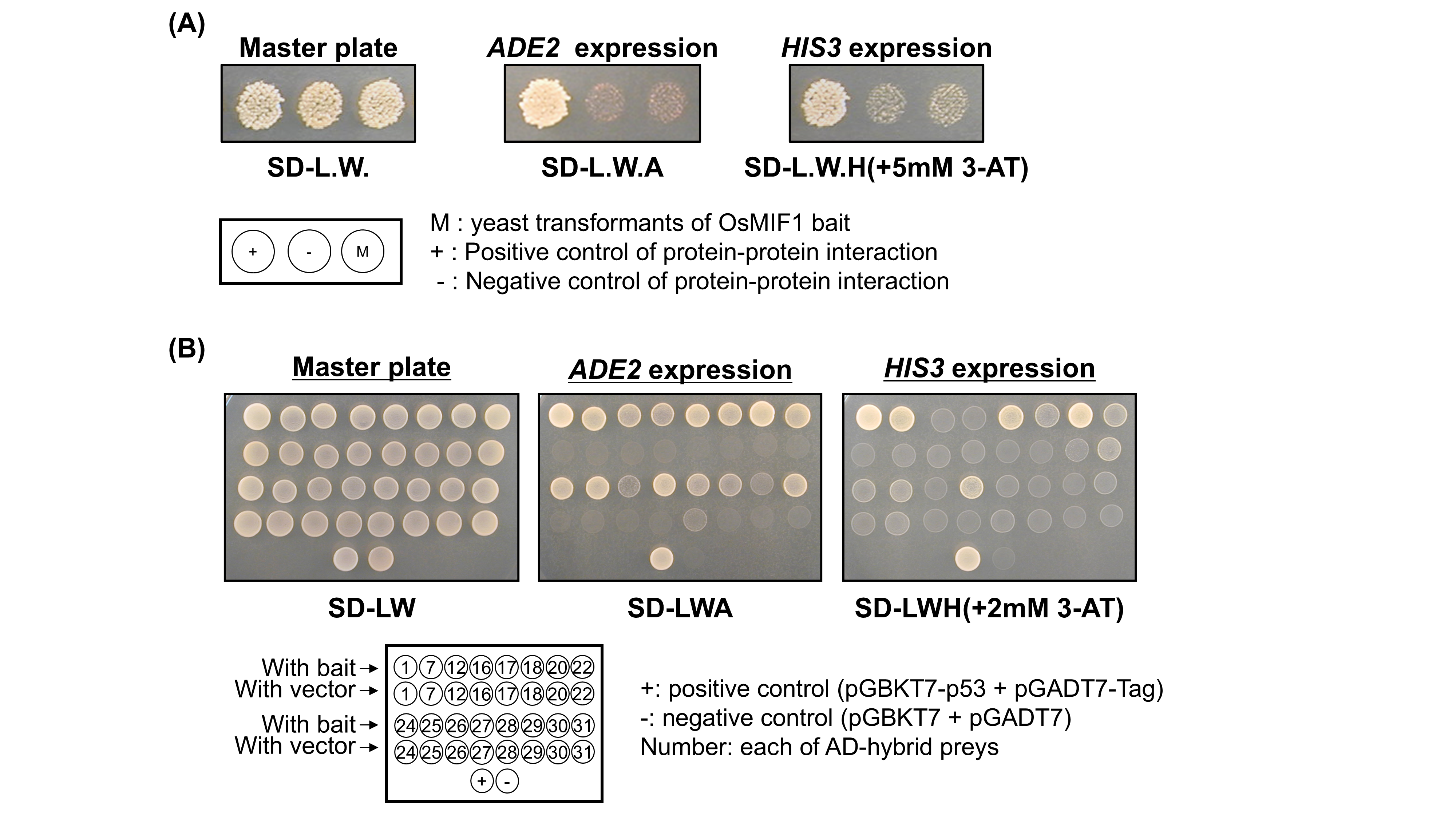


**Supplementary Fig. 15** Yeast two-hybrid (Y2H) library-scale screening. **(A)** Self-activation test of the OsMIF1 bait in the AH109 yeast strain. Yeast transformants containing OsMIF1 bait (M), a positive control (+), or a negative control (–) were grown on SD-LW master plates and assayed for ADE2 and HIS3 reporter gene activation on SD-LWA and SD-LWH (+5 mM 3-AT) media, respectively. **(B)** Y2H library-scale screening using OsMIF1 as bait. Yeast co-transformants containing OsMIF1 bait and each AD-hybrid prey clone were grown on SD-LW master plates and tested for ADE2 and HIS3 reporter gene activation on SD-LWA and SD-LWH (+2 mM 3-AT) media. Positive (+) and negative (–) controls are indicated, and numbers represent individual AD-hybrid prey clones.


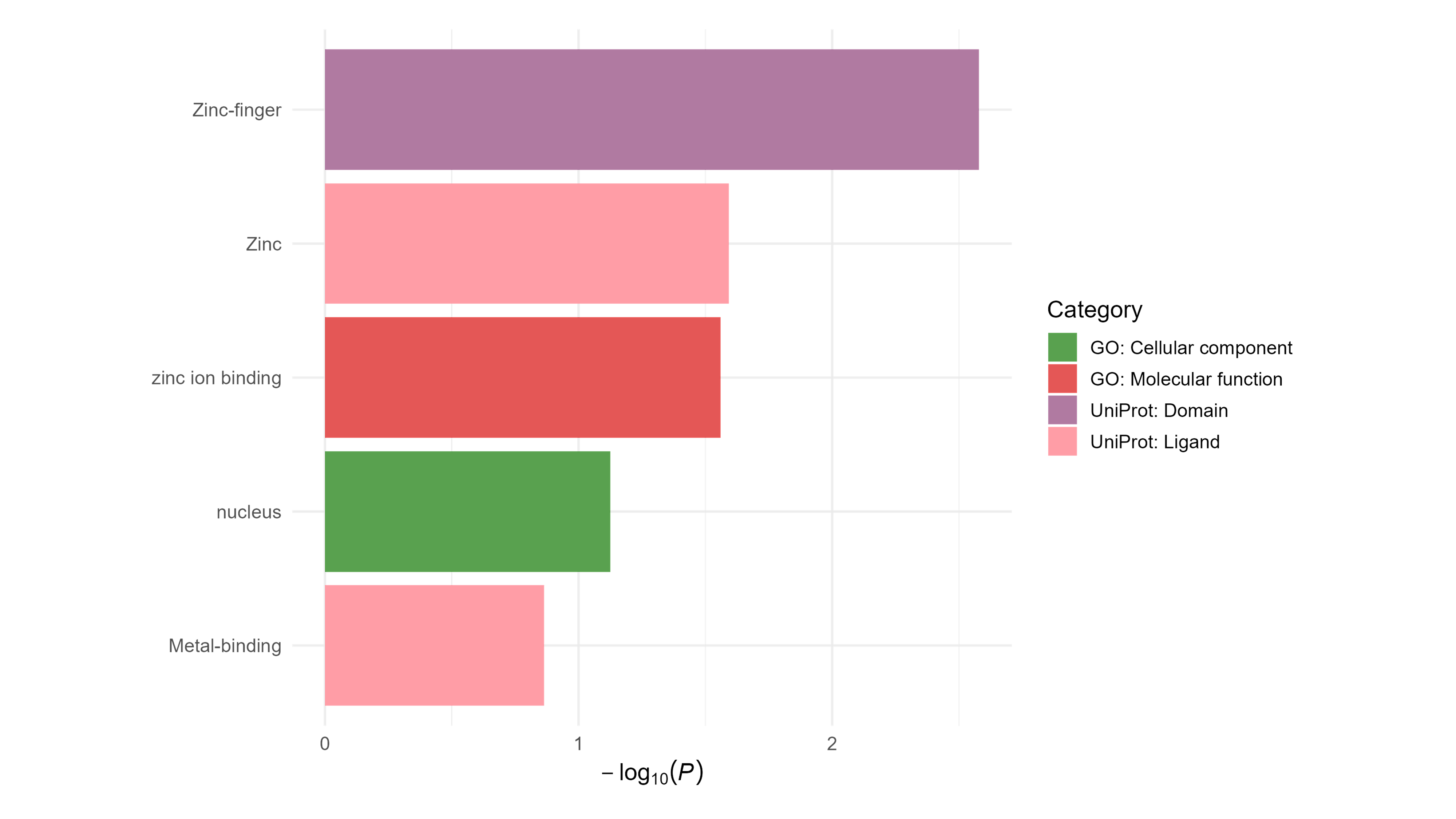


**Supplementary Fig. 16** Gene Ontology (GO) enrichment analysis of proteins identified from the Y2H library-scale screening with OsMIF1. GO enrichment analysis was performed using the DAVID tool (https://davidbioinformatics.nih.gov/).


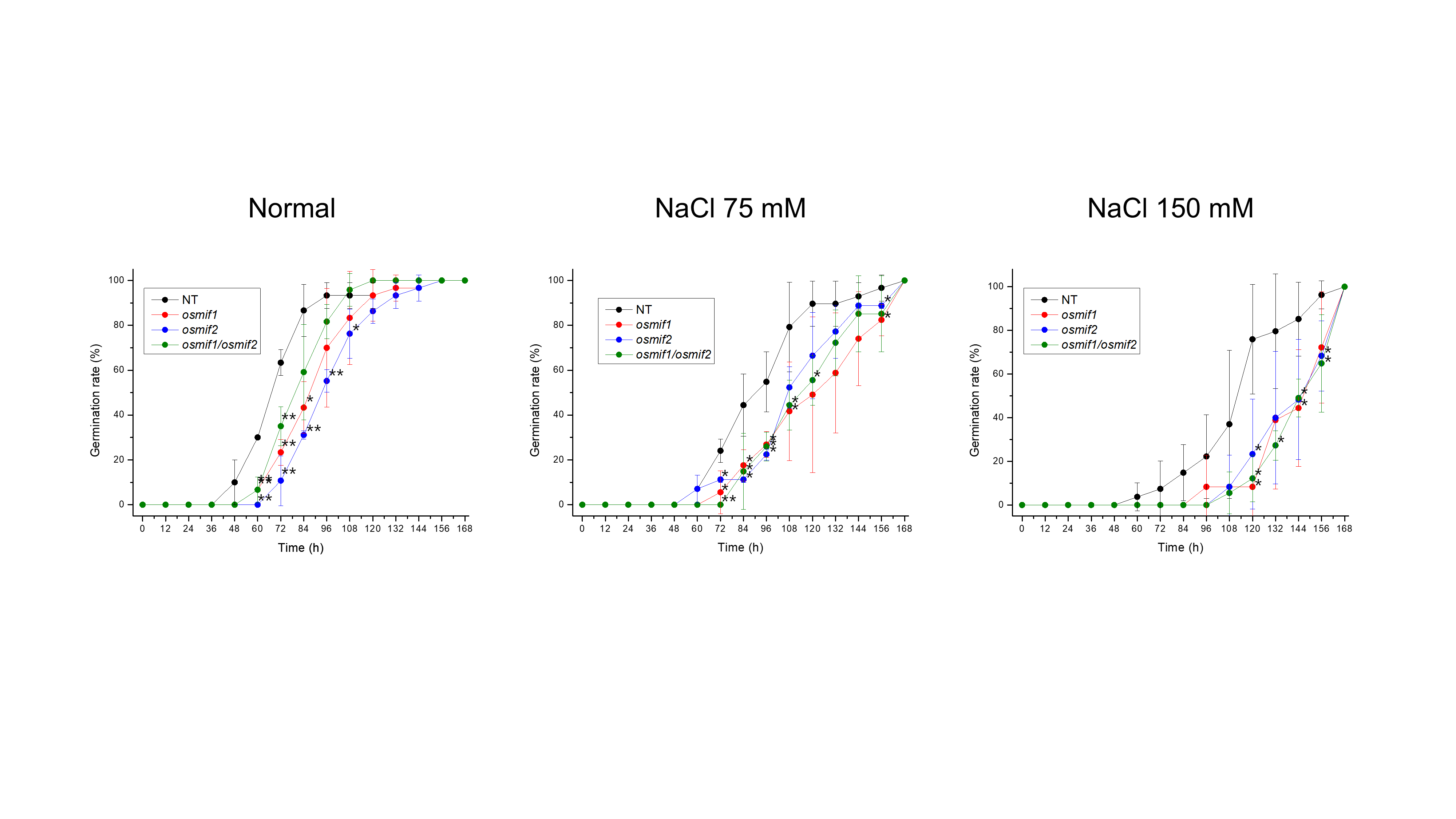


**Supplementary Fig. 17** Germination rates of OsMIF1 and OsMIF2 KO mutant lines under salt stress. Error bars represent SD (n = 30). *P*-values were calculated using Student’s t-test (*p< 0.1, **p< 0.01).
